# The Two Domains of the Avian Double-β-Defensin AvBD11 Have Different Ancestors, Common with Potential Monodomain Crocodile and Turtle Defensins

**DOI:** 10.3390/biology11050690

**Published:** 2022-04-30

**Authors:** Nicolas Guyot, Céline Landon, Philippe Monget

**Affiliations:** 1 INRAE, Université de Tours, BOA, F-37380 Nouzilly, France; 2 Centre de Biophysique Moléculaire, CNRS UPR 4301, F-45071 Orléans, France; celine.landon@cnrs-orleans.fr; 3 INRAE, CNRS, IFCE, Université de Tours, PRC, F-37380 Nouzilly, France; philippe.monget@inrae.fr

**Keywords:** AvBD11, avian double-beta-defensin, birds, reptiles, evolution, convergence, multiple sequence alignment, phylogenetic tree

## Abstract

**Simple Summary:**

Vertebrate defensins are a multigene family of antimicrobial peptides that evolved following a series of gene duplication and divergence events during the expansion of vertebrates. In birds, the repertoire of avian defensins contains an atypical defensin, namely AvBD11 (avian beta-defensin 11), which consists of two repeated but divergent defensin units (or domains) while most vertebrate defensins only possess one unit. In this study, we investigated the evolutionary scenario leading to the formation of this double defensin in birds by comparing each defensin unit of AvBD11 with other defensins from birds and closely related reptiles (crocodile, turtles) predicted to have a single defensin unit. Our most outstanding results suggest that the double defensin AvBD11 probably appeared following a fusion of two ancestral genes or from an ancestral double defensin, but not from a recent internal duplication as it can be observed in other types of proteins with domain repeats.

**Abstract:**

Beta-defensins are an essential group of cysteine-rich host-defence peptides involved in vertebrate innate immunity and are generally monodomain. Among bird defensins, the avian β-defensin 11 (AvBD11) is unique because of its peculiar structure composed of two β-defensin domains. The reasons for the appearance of such ‘polydefensins’ during the evolution of several, but not all branches of vertebrates, still remain an open question. In this study, we aimed at exploring the origin and evolution of the bird AvBD11 using a phylogenetic approach. Although they are homologous, the N- and C-terminal domains of AvBD11 share low protein sequence similarity and possess different cysteine spacing patterns. Interestingly, strong variations in charge properties can be observed on the C-terminal domain depending on bird species but, despite this feature, no positive selection was detected on the AvBD11 gene (neither on site nor on branches). The comparison of AvBD11 protein sequences in different bird species, however, suggests that some amino acid residues may have undergone convergent evolution. The phylogenetic tree of avian defensins revealed that each domain of AvBD11 is distant from ovodefensins (OvoDs) and may have arisen from different ancestral defensins. Strikingly, our phylogenetic analysis demonstrated that each domain of AvBD11 has common ancestors with different putative monodomain β-defensins from crocodiles and turtles and are even more closely related with these reptilian defensins than with their avian paralogs. Our findings support that AvBD11′s domains, which differ in their cysteine spacing and charge distribution, do not result from a recent internal duplication but most likely originate from a fusion of two different ancestral genes or from an ancestral double-defensin arisen before the Testudines-Archosauria split.

## 1. Introduction

Defensins are important components of innate immunity. Produced by a wide range of species, including vertebrates, invertebrates, plants, and fungi, these host-defence molecules are small cysteine-rich proteins (<10 kDa) with cationic properties for the most part. They have recently been classified into two phylogenetically distinct super-families, namely cis- and trans- [1,2], which ultimately explains why they differ in terms of cysteine motifs, disulphide bond connectivity, and 3D structures. In cis-defensins, the conserved CXC motif in the β-sheet orients the corresponding disulfide bridges in the same direction, whereas for trans-defensins the conserved vicinal cysteines (CC motif) in the β-sheet automatically orients the corresponding disulfides bridges in opposite directions. Defensins exhibit extremely diversified antimicrobial activities against various microorganisms including Gram+ and Gram- bacteria, fungi and enveloped viruses, to overcome the rapid evolution of pathogens. Moreover, immunomodulatory activities and other non-antimicrobial functions (e.g., enzyme inhibition, toxic function, anti-cancer activity, adaptation to abiotic stresses…) have also been reported for many defensins [1].

Vertebrate defensins belong to the trans-defensin super-family and are organized in a three-stranded antiparallel beta sheet with six conserved cysteines involved in three disulphide bridges that organize and stabilize the overall 3D structure. The β-defensin family is the most ancestral in vertebrate defensins and is present in all vertebrates (and even in some invertebrates) [1,3]. The α-defensin family, derived from the β-defensin family, is only found in mammals, while an additional small cyclic θ-defensin family, restrained to primates, is derived from the α-defensin family [3]. The rapid divergence of mammalian defensins following gene duplication events was found to be driven by positive selection [4,5].

In birds, only the β structural family is represented. Bird defensins are assembled in two divergent clusters of β-defensin genes within the genome: the avian β-defensins (AvBDs) cluster and the ovodefensins (OvoDs) cluster. Both clusters are present on chromosome 3 in the chicken genome. The AvBDs cluster (14 genes reported in chicken) is flanked by the CTSB and TRAM2 genes [6], while the OvoDs cluster (five genes in chicken) is surrounded by MTMR9 and XKR6 [7]. OvoDs, which are specific to reptiles and birds, have evolved to protect the cleidoic egg [8]. It is thought that OvoDs might have duplicated and diversified from AvBD12 [9].

**Figure 1:**
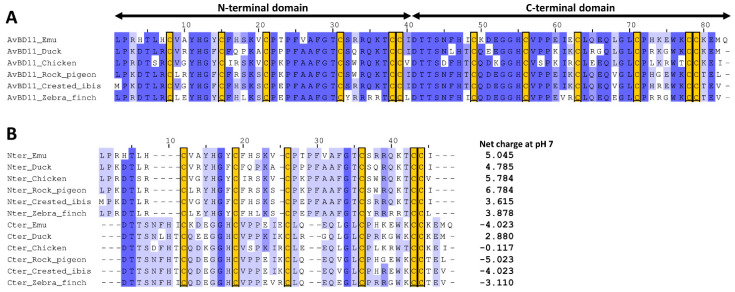
Multiple sequence alignments of AvBD11 and its two independent β-defensin domains in six evolutionarily distant birds (emu, duck, chicken, rock pigeon, crested ibis, zebra finch). (**A**) Mature AvBD11 protein sequences. (**B**) N-terminal (Nter) and C-terminal (Cter) domains of AvBD11. MSAs were performed with MAFFT and drawn with Jalview with a blue colour gradient (100% of identity for dark blue). Conserved cysteine residues are highlighted in yellow boxes.

Among bird β-defensins, AvBD11 is unique since it is the only AvBD possessing two β-defensin domains. The gene coding AvBD11 has been identified in at least 69 avian species, covering 32 out of the 40 orders of birds [10]. The two β-defensin domains are encoded by separate exons [10]. The chicken mature protein consists of 82 amino acids with a molecular weight of 9.3 kDa, and possesses 12 cysteines involved in six disulphide bonds [10]. Each domain adopts the three-stranded antiparallel β-sheet fold stabilized by the typical disulphide array C1-C5/C2-C4/C3-C6 of β-defensins. The spacing between cysteines follows the consensus pattern Cx_6_Cx_5_Cx_9_Cx_6_CCx_9_Cx_6_Cx_6_Cx_7_Cx_6_CC. The cysteine spacing patterns of each domain are distinct from monodomain AvBDs (Cx_4-7_Cx_3-4_Cx_9-10_Cx_5-6_CC) [6] but also from OvoDs (Cx_5_Cx_3_Cx_11_Cx_3_CC for OvoDA and Cx_3_Cx_3_Cx_11_Cx_4_CC for OvoDB) [8,11]. This double motif is highly conserved in AvBD11 within evolutionarily distant birds [10] (Figure 1A). AvBD11 is detected in the chicken egg, especially in the vitelline membrane [12,13] and in the egg albumen [14]. Accordingly, its expression is very high in the oviduct (organ of the female reproductive tract involved in egg formation) and is controlled by sex steroids [15]. AvBD11 is thought to play an important role in the antimicrobial defence of the avian egg and embryo and possibly other functions in avian reproduction. It possesses broad antimicrobial properties, previously demonstrated against Gram+ and Gram– bacteria, *Eimeria tenella* parasite and H1N1 influenza virus, as well as inhibitory effects on cell migration [10,16]. Unlike the antiviral activity which requires both domains of the protein in native form, the other biological activities are mainly carried by the N-terminal domain [10]. To date, the role of the C-terminal domain remains elusive.

The presence of defensins or defensin-like peptides with multiple β-defensin motifs (two or more) is rare in vertebrates. Intriguingly, some have been found in lizards (green anole [17], Gila monsters [18], Komodo dragon [19]), but not in other reptiles, such as crocodiles [20] or turtles [21]. To our knowledge, they have never been reported in mammals, and our search for such “polydefensins” in mammals has been unsuccessful. The Gila monster helofensin isoforms contain four defensin motifs that are thought to result from internal duplication events of an ancestral β-defensin that produced a single gene encoding a protein with four tandem repeated domains [18]. The reasons for the emergence of such ‘polydefensins’ during evolution in specific lineages of vertebrates and the functional gain over conventional ‘monodefensins’ are fascinating questions that remain unanswered.

**Figure 2:**
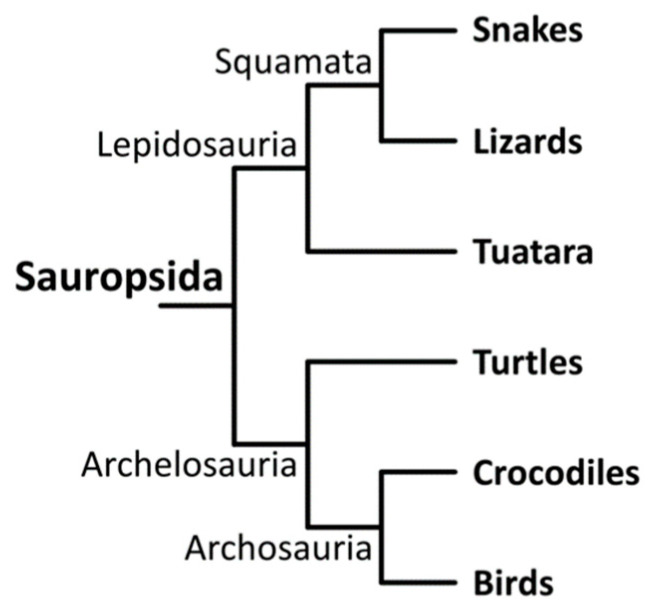
Cladogram of sauropsids. According to Crawford et al. [22] and Gemmell et al. [23].

The objective of this study is to investigate the origin and evolution of the avian double-domain defensin AvBD11 by studying the phylogenetic relationships between its two domains and putative reptilian β-defensins of closely related sauropsids. The construction of alignments and phylogenetic trees for highly variable and short sequences such as defensins is a challenge. This study was conducted using AvBD11 protein sequences of different avian species, including chicken, duck, crested ibis and zebra finch, that belong to distinct bird orders. The phylogenetic analysis was carried out within birds (Aves), crocodiles and turtles (Testudines). A cladogram of sauropsids showing the position of birds in this clade is shown in Figure 2.

## 2. Materials and Methods

### 2.1. Protein Sequences and Net Charge Determination

AvBD11 protein sequences from emu (*Dromaius novaehollandiae*, XP_025969281.1), duck (*Anas platyrhynchos*, XP_005028303.2), chicken (*Gallus gallus*, NP_001001779.1), rock pigeon (*Columbia livia*, XP_005513695.1), crested ibis (*Nipponia nippon*, XP_009465634.1), and zebra finch (*Taeniopygia guttata*, XP_002186664.2) were retrieved from the NCBI (National Center for Biotechnology Information) protein database. Mature sequences were determined using the SignalP 5.0 server (https://services.healthtech.dtu.dk/service.php?SignalP-5.0, accessed 1 February 2022). Sequences corresponding to the N-terminal and C-terminal domains of mature AvBD11 were defined according to a previous study on chicken AvBD11 [10]. Separate domains of AvBD11 were used for multiple sequence alignments and phylogenetic analyses. Net charges of peptides at pH 7 were calculated with Prot Pi Peptide Tool (https://www.protpi.ch/Calculator/PeptideTool, accessed 1 December 2021) using ‘Expasy’ as data source of pKa value.

All other AvBD sequences from chicken (*Gallus gallus*), duck (*Anas platyrhynchos*), crested ibis (*Nipponia nippon*), and zebra finch (*Taeniopygia guttata*) used for phylogenetic analyses were retrieved from a published work [6]. OvoD sequences from these species were obtained from two previous studies [7,8]. Beta-defensin sequences from four crocodile species (*Alligator mississippiensis*, American alligator; *Alligator sinensis*, Chinese alligator; *Crocodylus porosus*, saltwater crocodile; *Gavialis gangeticus*, gharial) were taken from a recent work [20]. Putative β-defensin sequences from turtle species (*Chrysemys picta bellii*, western painted turtle; *Chelydra serpentina*, common snapping turtle; *Pelusios castaneus*, West African mud turtle; *Terrapene Carolina triunguis*, three-toed box turtle) were retrieved from annotated genes in the Ensembl database. Mature protein sequences were obtained using the SignalP 5.0 server, as previously described. All acronyms (for animal species) and protein sequences used in phylogenetic trees are summarized in Tables S1 and S2 (Supplemental Data), respectively.

### 2.2. Multiple Sequence Alignment (MSA)

All protein alignments were performed in the Jalview 2 desktop application [24] with different subsets of defensin sequences depending on the purpose. In particular, AvBDs sequences including separate domains of AvBD11 were aligned with OvoDs, crocodile defensins or turtle defensins in phylogenetic analyses to investigate phylogenetic relationships within birds, between birds and crocodile, or between birds and turtles, respectively. Multiple sequence alignments are the essential first step in studying molecular phylogeny. To produce an alignment of a set of sequences with low identity, the automated alignment is often followed by a manual adjustment. Here, most of the multiple sequence alignments were automatically performed with the program MAFFT (Multiple Alignment using Fast Fourier Transform) [25] using the default parameters (BLOSUM62 matrix and gap penalty 1.53). Note that, to ensure alignment of the structurally conserved cysteine residues without any additional manual adjustments when crocodile sequences are aligned with bird sequences, the standard parameters need to be optimized (BLOSUM40 matrix and gap penalty 1.53.

### 2.3. Construction of Phylogenetic Trees

Phylogenetic trees were constructed using the web service Phylogeny.fr [26] with the ‘à la carte’ mode (http://www.phylogeny.fr/alacarte.cgi, accessed 1 December 2021). The methodological approach used to construct trees with β-defensins is discussed in the Results and discussion section. Previously generated MSAs (untreated or trimmed to adjust to the length of N-terminal and C-terminal AvBD11) were submitted to a workflow containing the tree construction (PhyML) and visualisation (TreeDyn) steps, preceded or not with an automatic curation step (removal of positions with gaps). Default parameters were used. The program PhyML, used to construct the trees, is based on the maximum-likelihood principle [27]. The scale represents the substitution rate. Only trimmed MSAs and corresponding trees (without automatic curation) are presented in the main article. Full (untreated) MSAs and corresponding trees (with or without automatic curation) are shown in Supplemental Data (Figures S1–S6). For the construction of trees, we have used the approximate likelihood-based measures of branch supports (aLRT, approximate likelihood ratio test), shown to provide a compelling alternative to slower conventional methods and which offers excellent levels of accuracy and power [28]. The branches are supported by an aLRT-branch support value.

### 2.4. Positive Selection Calculation

Positive selection was calculated as previously described [29,30]. The inference of positive selection was performed on the tree of AvBD11 genes from thirty avian species by balancing the species according to the charge properties of the C-terminal domain with branch-site and site models of codeml of the PAML (Phylogenetic Analysis Using Maximum Likelihood) package [31]. This analysis was carried out on the coding-DNA sequence (CDS) region corresponding to mature AvBD11. Thirty different species were selected with regard to the net charge of the C-terminal domain (balanced number of sequences with acidic, neutral or basic C-terminal domains). All nucleotide sequences used in this study are shown in Table S3 (Supplemental Data). The MSA was carefully examined to avoid all false positive results. In particular, amino acids predicted to be under positive selection that were at the boundary of the alignments were not considered because they are doubtful. Both branch-site and site models are designed to identify amino acids under positive selection. However, the site model allows the ω ratio (dN/dS ratio, i.e., the ratio of non-synonymous mutations (dN) in the coding sequence to synonymous mutations (dS)) to vary among sites, i.e., among amino acids in the protein. The branch-site model on the other side allows ω to vary among sites in the protein and across branches on the tree and therefore aims to detect positive selection affecting a few sites along particular lineages, sites that would not be detected by using the site model. For the use of branch-site models, each branch of the phylogenetic tree was tested for positive selection. We performed multiple test corrections by controlling for the false discovery rate (FDR) using the R package QVALUE [32]. Results are considered significant with a threshold of q = 10% of false positives. Sites with posterior probabilities of Bayes empirical Bayes analyses superior to 95% or 99% were considered as positively selected. No overlap was found between the two models because the branch-site model is detecting positive selection on a selected branch, while the site model is detecting positive selection affecting the whole phylogenetic tree.

### 2.5. Searching for Homolog Sequences of AvBD11 in Non-Avian Sauropsids

Homolog sequences of chicken AvBD11 were retrieved using the BLAST (Basic Local Alignment Search Tool) search tool in Ensembl (https://www.ensembl.org/Multi/Tools/Blast, accessed 1 December 2021) and various query sequences: mature chicken AvBD11 or corresponding domains (sequences are shown in Figure 1). BLASTp was run with the BLOSUM62 matrix and an E-value threshold of 1 × 10^−3^ against a panel of various available species of non-avian sauropsids, including turtles/tortoises (*Chelonoidis abingdonii*, *Chelydra serpentina*, *Chrysemys picta bellii*, *Gopherus agassizii*, *Gopherus evgoodei*, *Pelodiscus sinensis*, *Pelusios castaneus*, *Terrapene Carolina triunguis*), snakes (*Laticauda laticaudata*, *Naja naja*, *Notechis scutatus*, *Pseudonaja textilis*), lizards (*Anolis carolinensis*, *Podarcis muralis*, *Pogona vitticeps*, *Salvator merianae*, *Varanus komodoensis*), the tuatara (*Sphenodon punctatus*), and a crocodile (*Crocodylus porosus*). tBLASTn was conducted with the BLOSUM62 matrix and an E-value threshold of 1 × 10^−1^ on the same panel of species.

## 3. Results and Discussion

### 3.1. Degree of Divergence of the N- and C-Terminal Domains of AvBD11

The double-β-defensin AvBD11 is highly conserved in birds [10]. The representative alignment of mature AvBD11 protein sequences in six evolutionarily distant birds belonging to different orders (Casuariiformes, Anseriformes, Galliformes, Columbiformes, Pelecaniformes, Passeriformes) shown in Figure 1A attests to the high identity of protein sequences and the conservation of the cysteines involved in the two β-defensin folds. In spite of obvious homology, a divergence in the cysteine spacing was previously observed between the two domains [10]. To investigate whether AvBD11 has arisen from an internal duplication of an ancestral gene or exon, as previously assumed for the Gila monster helofensin isoforms [18], the two defensin domains of AvBD11 were first aligned to better appreciate their degree of identity. Knowing that the AvBD11 β-defensin motifs are encoded by two independent exons [10], the full-length mature AvBD11 sequences used in Figure 1A were split at the position corresponding to the exon-exon junction and aligned in Figure 1B. The multiple sequence alignment of N-terminal and C-terminal domains in Figure 1B shows that the two defensin domains of AvBD11 share a low sequence identity, which is in contrast with the strong conservation of mature AvBD11 sequences observed between species. Note that the cysteine spacing patterns are different in both domains (Cx_6_Cx_5_Cx_9_Cx_6_CC and Cx_6_Cx_6_Cx_7_Cx_6_CC for N- and C-terminal domains, respectively), as previously observed [10]. As a consequence, gaps are formed in the MSA between C2 and C3 for N-terminal domains and between C3 and C4 for C-terminal domains (Figure 1B). Importantly, the cysteine spacing patterns of each domain of AvBD11 are distinct from monodomain AvBDs [6] but also from OvoDs [8,11]. Besides their poor sequence identity, N- and C-terminal domains differ in the presence of acidic amino-acids (Glu and Asp) being more abundant in the C-terminal domain (e.g., 8 in emu, 4 in duck, and 7 in zebra finch) than in the N-terminal domain (0, 1, and 3, respectively) (Figure 1A,B). Interestingly, the calculated net charge (at pH 7) of AvBD11 domains are differentially conserved as a function of bird species. The N-terminal domain is indeed positively charged in all six considered species, such as most vertebrate defensins and antimicrobial peptides, while net charges of the C-terminal domain are either positive (duck), neutral (chicken), or negative (emu, rock pigeon, crested ibis, zebra finch) (Figure 1B). It is likely that such variations in charge properties may have an impact on the biological function of AvBD11 within bird species. In this respect, the two domains of chicken AvBD11 appeared to functionally differ regarding the antibacterial properties [10], but the contribution of charges (number, localization) in this feature still remains to be clearly defined. Altogether, these findings support the fact that the N- and C-terminal β-defensin domains of AvBD11 highly differ from each other from a structural and functional point of view.

### 3.2. Analysis of Positive Selection and Detection of Amino Acids under Potential Convergent Evolution in AvBD11 Sequences

In the coding sequence of a gene, synonymous mutations are assumed to be neutral, and non-synonymous mutations are assumed to be generally deleterious and thus purged from the populations, except if it represents an advantage for individuals. There are two types of natural selection in biological evolution: negative or purifying selection which is observed when the ratio ω of non-synonymous mutations (dN) in the coding sequence to synonymous mutations (dS) is less than 1, and positive selection when this dN/dS ratio is greater than 1. The rapid divergence of mammalian β-defensins following gene duplication events was previously found to be driven by positive selection [4,5]. Positive selection is thought to have also occurred in reptilian and avian β-defensins [6,20]. A previous study on avian β-defensins demonstrated that the effect of such selection, however, was very weak in AvBD9, AvBD11, and AvBD13 [6]. Due to the numerous AvBD11 sequences available in databases, we performed a selection analysis on a limited number of sequences. Thirty different species were selected with regard to the charge of the C-terminal domain (balanced number of sequences with acidic, neutral, or basic C-terminal domains). In the present study, we did not detect a positive selection signal on the mature AvBD11 sequences, neither with the site model nor with the branch-site model, even after using several models (the phylogenetic tree with omega values for the branch-site model is presented in Figure S7, Supplemental Data). Therefore, the evolution of AvBD11 in these bird species is not likely to be driven by positive selection. One could have imagined that adaptive selection would generate different strategies for the mode of action of this protein between species as observed for other families, for example in the case of genes which encode the proteins of immunity in birds [33] or even as we have shown for genes encoding the odorant binding proteins (OBP) [34] or the receptors of the melatonin [35].

Based on a previously published MSA of mature AvBD11 from various avian species [10], it seems that some amino acids in both domains may have undergone evolutionary convergence, since they are divergent between closely related species in the tree of life, but identical between very distant bird species in the tree of life. As shown in Figure S8 (Supplemental Data), these substitutions can have relatively minor consequences (Arg/Lys at position 3, Phe/Tyr at position 14, according to the mature chicken AvBD11 numbering) or can be associated with dramatic modifications of polarity and/or charge properties (Arg/Trp at position 33, Glu/Lys/Gly at position 75). Interestingly, the latter substitutions observed at positions 33 and 75 are located in loop regions (see 3D structure of chicken AvBD11 in Figure S9, Supplemental Data), which are usually considered as the most variable structural elements to modulate/modify protein functions [36].

### 3.3. Methodological Approach Used to Construct Phylogenetic Trees with β-Defensins

The evolution of AvBD11 domains was then further assessed among the diversity of avian and reptilian defensins sequences using a phylogenetic approach. On the assumption that all of the aligned sequences are β-defensins (thus sharing the common disulphide bridges array described above, but with variable cysteine spacings), attention was paid here to favour the alignment of cysteines in the MSAs at the cost of gap formations when needed. A curation step is usually performed to clean up the MSA by removing gaps and/or variable regions, prior to the construction of phylogenetic trees. Insertions/deletions creating gaps in MSAs are often considered to be problematic in molecular phylogenetics [37]. For this reason, most phylogenetic studies treat gaps as missing data or remove gap columns from the MSA. However, for short sequences such as defensins, with highly variable regions and variations of cysteine spacings, MSA ineluctably induces gaps. In such cases, the use of a curation step might be an issue for the phylogenetic accuracy, since this step may considerably shorten the lengths of initial sequences. Therefore, removing gaps can be detrimental with this family of proteins. Several studies argue that gapped regions contain substantial phylogenetic signal that contributes to the accuracy of reconstructed trees [38,39,40]. It is also noteworthy that, even with standard alignment and tree building methods, excluding gaps and variable regions can worsen the resulting trees [40]. Consequently, we opted for constructing trees from non-curated MSAs and checked the results obtained with automatic curation (removal of gappy columns) (Supplemental Data, Figures S2B, S4B and S6B). Moreover, it should be noted that the length of the N- and C-terminal ends on both sides of the cysteine core can vary depending on defensins, even in mature forms. Given the short size of the cysteine core, we assume that these variable regions may considerably influence the accuracy of trees. Therefore, MSAs were manually trimmed by adjusting the length to the AvBD11 domains prior to the submission to the tree construction program. Thus, we chose to show these MSAs in the main text, and the resulting trees obtained without gap removal, which actually represent an intermediate condition between full (Supplemental Data, Figures S1, S3 and S5) and automatically curated (gap positions removed) MSAs, for the construction of trees.

### 3.4. Phylogeny of N- and C-Terminal Domains of AvBD11 among AvBDs and OvoDs

**Figure 3:**
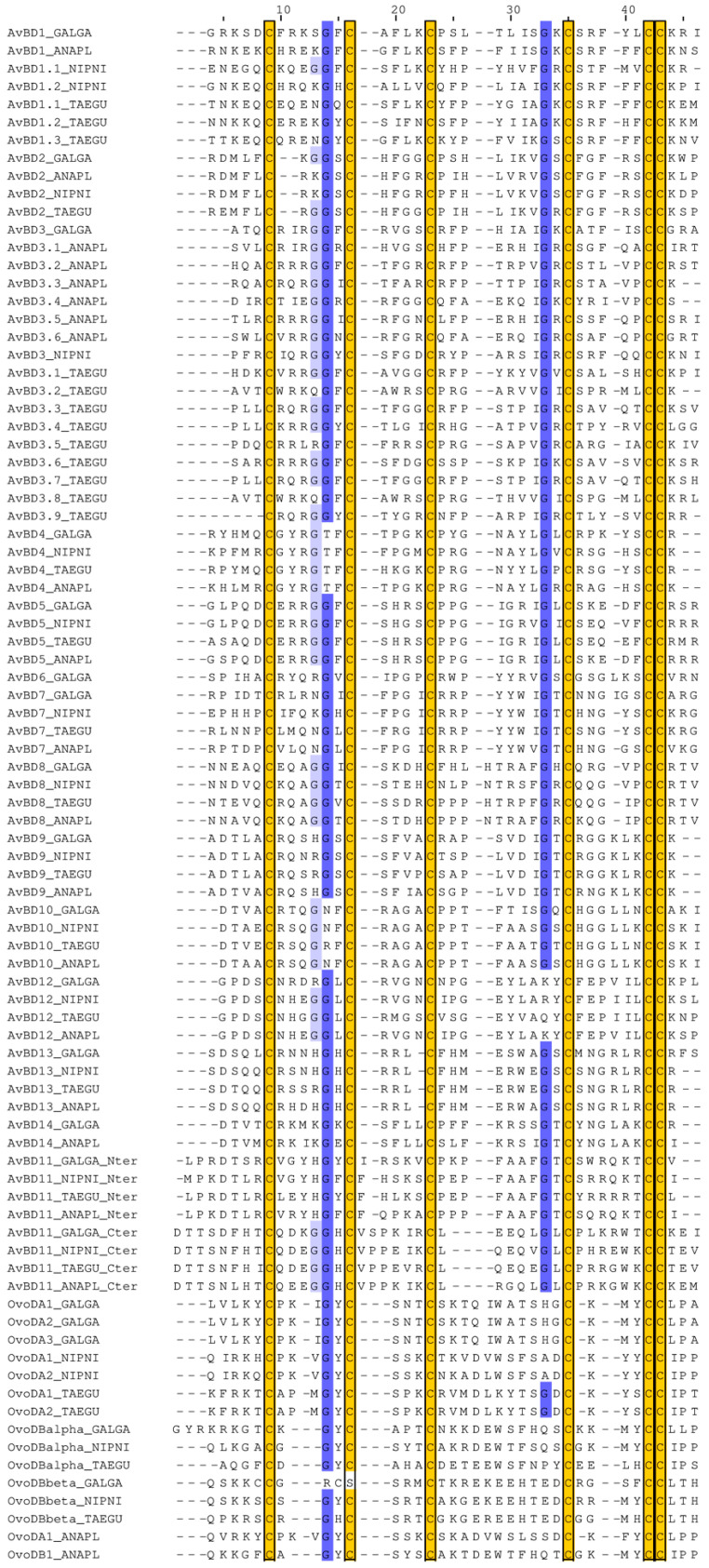
MSA of N-terminal and C-terminal domains of AvBD11 with monodomain AvBDs and OvoDs. MSA was performed with MAFFT and drawn with Jalview with a blue colour gradient (100% of identity for dark blue) and trimmed at each extremity to adjust to the length of the N-terminal and C-terminal domains of AvBD11. Conserved cysteine residues are highlighted in yellow boxes. ANAPL, *Anas platyrhynchos* (duck); GALGA, *Gallus gallus* (chicken); NIPNI, *Nipponia nippon* (crested ibis); and TAEGU, *Taeniopygia guttata* (zebra finch).

**Figure 4:**
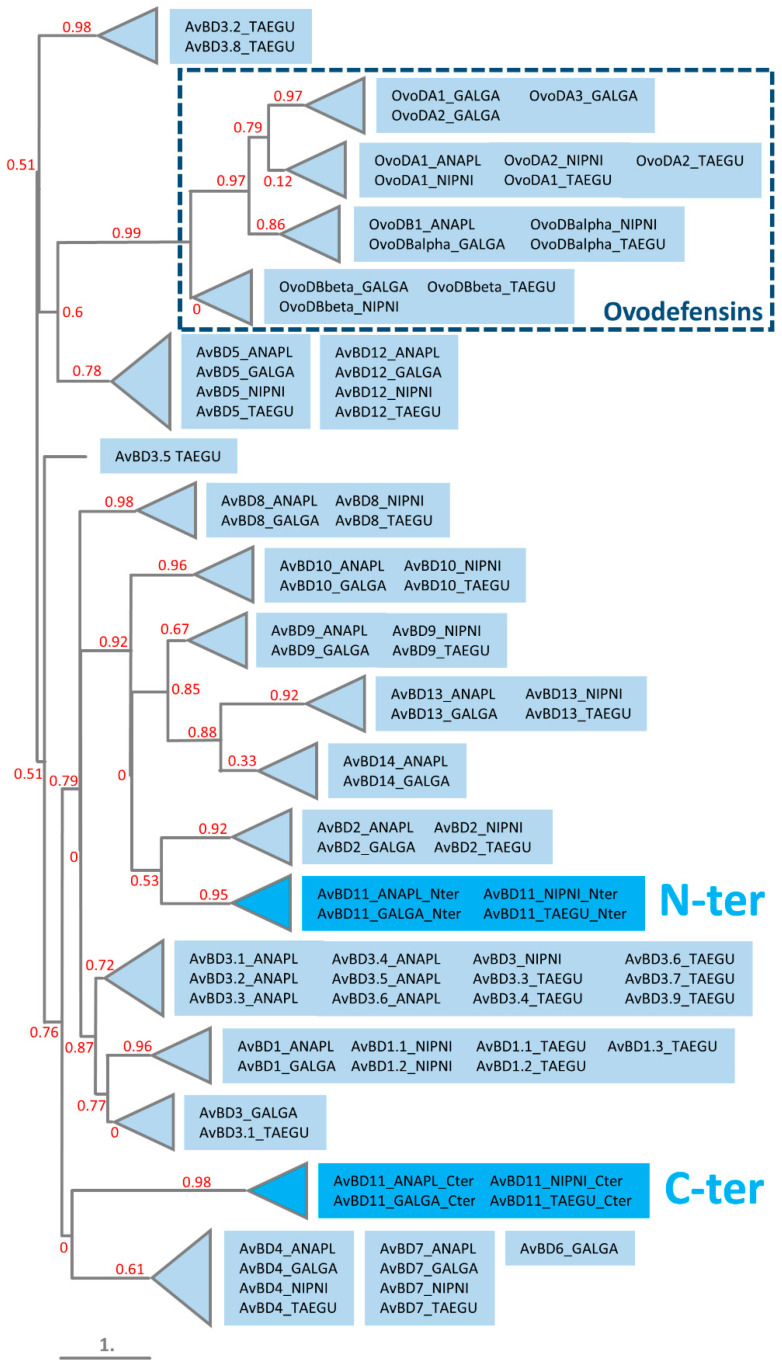
Phylogenetic relationships of the N-terminal (N-ter) and C-terminal (C-ter) domains of AvBD11 with monodomain AvBDs and OvoDs in four distant birds (duck, chicken, crested ibis, zebra finch). The phylogenetic tree was constructed using the maximum-likelihood-based program PhyML from MAFFT-based alignments adjusted to the length of N-ter and C-ter AvBD11. Branch support values are indicated in red. ANAPL, *Anas platyrhynchos* (duck); GALGA, *Gallus gallus* (chicken); NIPNI, *Nipponia nippon* (crested ibis); and TAEGU, *Taeniopygia guttata* (zebra finch).

The MSA and the phylogenetic tree of monodomain AvBDs (AvBD11 split into N-terminal and C-terminal domains) and OvoDs in four evolutionarily distant bird species (duck, chicken, crested ibis, zebra finch) are shown in Figure 3 and Figure 4, respectively. In the tree presented in Figure 4, all of the OvoDs are clustered together and branched with AvBD5 and AvBD12, forming a group isolated from all the remaining AvBDs. Of note, in trees with full and gap-free MSAs, OvoDs appear either associated with AvBD6/AvBD7 (Figure S2A in Supplemental Data) or completely isolated (Figure S2B in Supplemental Data). Regardless of the MSAs used to construct trees, our results reveal that the two domains of AvBD11 are well differentiated from the OvoDs and are divergent from each other. In Figure 4, the C-terminal is not closely clustered with any other AvBDs (the relationship with AvBD4/AvBD6/AvBD7 is supported with a null branch support value), while the N-terminal domain seems to have a common ancestor with several AvBDs, including AvBD2, AvBD9, AvBD10, AvBD13, and AvBD14. These results are rather consistent with the tree obtained with the full MSA without curation (Figure S2A in Supplemental Data) but not with that obtained after the removal of gappy columns (Figure S2B in Supplemental Data). The tree results concerning AvBD11 domains in Figure 4 are similar to a previously published phylogenetic tree showing that the N- and C-terminal domains of AvBD11 are related to AvBD9/AvBD10/AvBD14 and AvBD4, respectively [41]. In most MSAs available in the literature, monodomain AvBDs are aligned with the N-terminal domain of AvBD11 when full-length AvBD11 (with both domains) is used. Our findings are in agreement with most published phylogenetic studies on AvBDs, showing that AvBD11 is primarily related to either AvBD9 [42,43,44,45,46,47], AvBD10 [6,48], or AvBD13/AvBD14/AvBD9 [49], while some publications rather reveal relationships with AvBD5 [50], AvBD8 [51], or AvBD5/AvBD4/AvBD8/AvBD10/AvBD1/AvBD2 [52]. The type of sequences (nucleotide, protein) and the methods used to build up the alignments and trees may explain, in part, the discrepancies of these data. In the present study, we used the MAFFT program for the MSA, which is known to be more accurate than ClustalW [53] used by other authors [52]. Taken together, our results strongly support that each domain of AvBD11 is distant from OvoDs and may have arisen from different ancestral defensins.

### 3.5. Phylogeny of N- and C-Terminal Domains of AvBD11 among AvBDs and Crocodile Defensins

**Figure 5:**
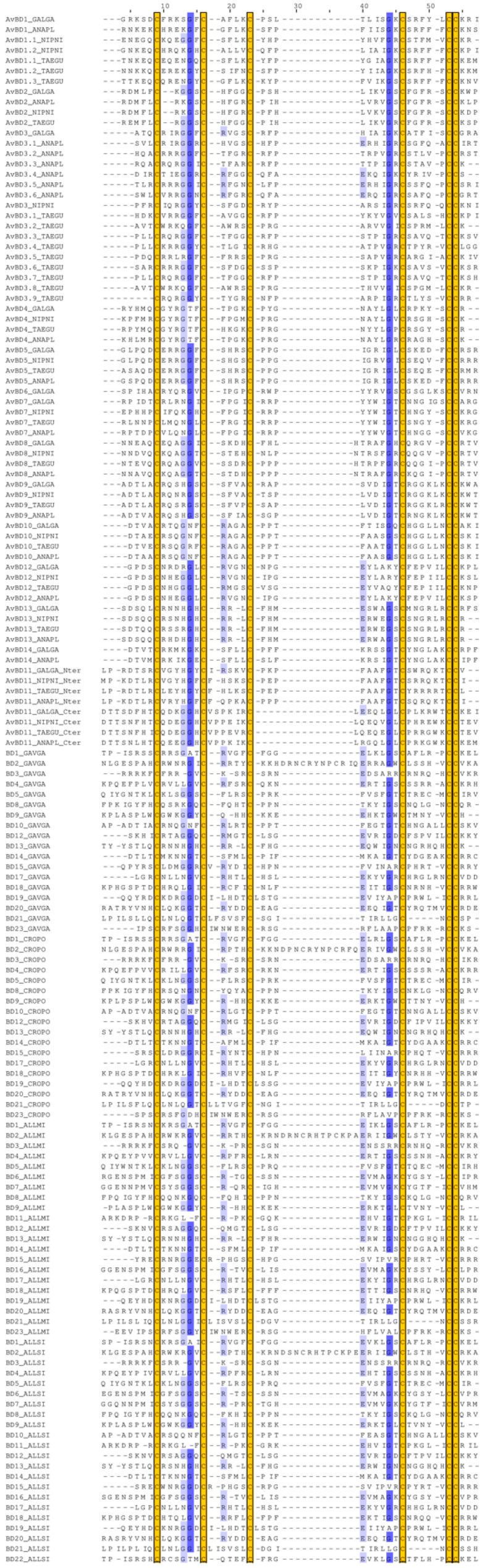
MSA of the N-terminal and C-terminal domains of AvBD11 with monodomain AvBDs and crocodile β-defensins. MSA was performed with MAFFT and drawn with Jalview with a blue colour gradient (100% of identity for dark blue) and trimmed at each extremity to adjust to the length of the N-terminal and C-terminal domains of AvBD11. Conserved cysteine residues are highlighted in yellow boxes. Bird species acronyms: ANAPL, *Anas platyrhynchos* (duck); GALGA, *Gallus gallus* (chicken); NIPNI, *Nipponia nippon* (crested ibis); and TAEGU, *Taeniopygia guttata* (zebra finch). Crocodile species acronyms: ALLMI, *Alligator mississippiensis* (American alligator); ALLSI, *Alligator sinensis* (Chinese alligator); CROPO, *Crocodylus porosus* (saltwater crocodile); and GAVGA, *Gavialis gangeticus* (gharial).

**Figure 6:**
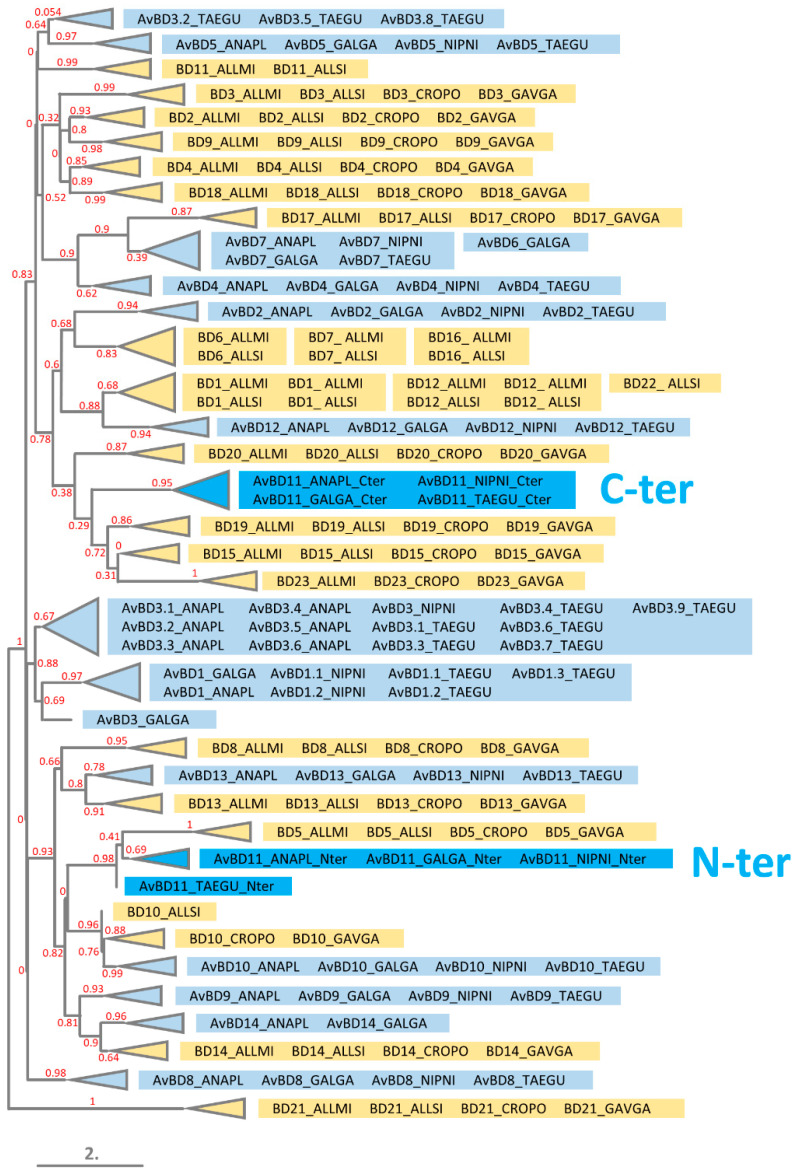
Phylogenetic relationships of the N-terminal (N-ter) and C-terminal (C-ter) domains of AvBD11 with monodomain AvBDs and crocodile beta-defensins. The phylogenetic tree was constructed using the maximum-likelihood-based program PhyML from MAFFT-based alignments adjusted to the length of N-ter and C-ter AvBD11. Branch support values are indicated in red. Bird and crocodile β-defensins are highlighted in blue and in yellow, respectively. Bird species acronyms: ANAPL, *Anas platyrhynchos* (duck); GALGA, *Gallus gallus* (chicken); NIPNI, *Nipponia nippon* (crested ibis); and TAEGU, *Taeniopygia guttata* (zebra finch). Crocodile species acronyms: ALLMI, *Alligator mississippiensis* (American alligator); ALLSI, *Alligator sinensis* (Chinese alligator); CROPO, *Crocodylus porosus* (saltwater crocodile); and GAVGA, *Gavialis gangeticus* (gharial).

Crocodiles are the closest relatives of birds in the Sauropsida clade (Figure 2). Santana and colleagues recently characterized a cluster of putative β-defensin-coding genes in the genomes of four crocodilian species, namely the saltwater crocodile (*Crocodylus porosus*), the American alligator (*Alligator mississipiensis*), the Chinese alligator (*Alligator sinensis*), and the gharial (*Gavialis gangeticus*) [20]. Therefore, we explored eventual phylogenetic relationships of β-defensins between birds and crocodiles. Interestingly, in crocodiles, the β-defensin cluster is flanked by CTSB and TRAM2 genes and is then syntenic with the AvBDs cluster in chicken [6]. It is also noteworthy that only monodomain defensin genes were reported in Santana’s work. However, in the Ensembl database, the ENSCPRG00005002116 gene from *Crocodylus porosus* is predicted to encode two putative transcripts, one of them being a potential double-defensin that actually corresponds to a fusion of BD12 and BD13 sequences in Santana’s study. The MSA and the phylogenetic tree of monodomain AvBDs (including split AvBD11) and crocodile β-defensins are shown in Figure 5 and Figure 6, respectively.

The tree in Figure 6 shows that the N-terminal domain preferentially associates with crocodile BD5 and more distantly with BD10/AvBD10, BD14/AvBD14, and AvBD9. The C-terminal domain of AvBD11 appears to be related with crocodile BD15, BD19, and BD23 but with low confidence (poor branch support values). These results are consistent with the full MSA-based tree (Supplemental Data, Figure S4A), where the relationships for each domain are supported with reliable branch support values. Remarkably, the two domains of AvBD11 appear in two different clusters and preferentially cluster with crocodile monodomain defensins, not bird defensins (Figure 6 and Figure S4A). Of note, other AvBDs also cluster with crocodile defensins rather than AvBDs: AvBD2 and BD6/BD7/BD16 (0.68 branch support value), AvBD7 and BD17 (0.9 branch support value), AvBD10 and BD10 (0.96 branch support value), AvBD12 and BD1/BD12/BD22 (0.88 branch support value), AvBD13 and BD13 (0.8 branch support value), AvBD14 and BD14 (0.9 branch support value) (Figure 6). It seems that each domain of AvBD11 has common ancestor(s) with different crocodile monodomain defensins. Altogether, these findings support our hypothesis that the two domains of AvBD11 do not result from an internal duplication but rather from two different ancestral genes.

### 3.6. Identification of Reptilian Homologs for the N-Terminal and C-Terminal Domains of AvBD11

**Figure 7:**
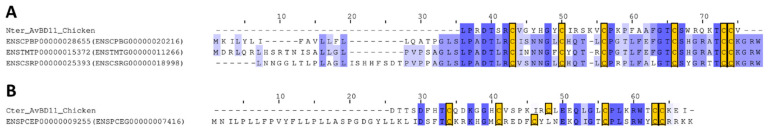
Alignments of the N-terminal (**A**) and C-terminal (**B**) domains of chicken AvBD11 with turtle homologs identified by BLAST. The MSA was performed with MAFFT and drawn with Jalview with a blue colour gradient (100% of identity for dark blue). Conserved cysteine residues are highlighted in yellow boxes.

Closely related homologs of AvBD11 were tentatively searched in the Ensembl database within the class of Reptilia (clade of Sauropsida without Aves). Mature chicken AvBD11 protein and isolated domain sequences were analysed by BLAST in the Ensembl server with an E-value threshold of 1 × 10^−3^. All of the species selected for this BLAST search are indicated in the Materials and methods section. Surprisingly, only hits associated with turtle/tortoise sequences were found. Full-length and N-terminal chicken AvBD11 sequences indeed gave three hits, including translation IDs ENSCSRP00000025393 from common snapping turtle *Chelydra serpentina* (Gene: ENSCSRG00000018998, E-value ≤ 2 × 10^−6^), ENSCPBP00000028655 from painted turtle *Chrysemys picta bellii* (Gene: ENSCPBG00000020216, E-value ≤ 3 × 10^−4^), and ENSTMTP00000015372 from three-toed box turtle *Terrapene carolina triunguis* (Gene: ENSTMTG00000011266, E-value ≤ 6 × 10^−5^). One hit was found with the C-terminal chicken AvBD11: ENSPCEP00000009255 from West African mud turtle *Pelusios castaneus* (Gene: ENSPCEG00000007416, E-value: 8 × 10^−4^). Similar hits were obtained with tBLASTn at a threshold of 1 × 10^−1^ using full-length and N-terminal chicken AvBD11 sequences (no hits with the C-terminal chicken AvBD11 sequence). All of these sequences obtained with BLASTp and tBLASTn contain the typical β-defensin consensus sequence that mainly fit either with the N-terminal or with the C-terminal domain of AvBD11, as shown in the alignments presented in Figure 7.

Despite the similarity between these bird and turtle sequences, a gap is observed between the second and the third Cys in the alignment with the N-terminal AvBD11 (Figure 7A), and the third Cys between ENSPCEP00000009255(ENSPCEG00000007416) and the C-terminal AvBD11 is misaligned (Figure 7B). Very strikingly, no hits related to the saltwater crocodile *Crocodylus porosus* were retrieved with BLASTp and tBLASTn in our threshold conditions nor BD5 (ENSCPRG00005002069) or BD15/BD19/BD23, which we found to be related with the N-terminal and C-terminal domains of AvBD11, respectively. Counterintuitively, although crocodiles are the closest relatives of birds (Figure 2), our results may indicate that the sequences of the two domains of AvBD11 are more closely related to turtle defensins than to those of crocodiles. No hits were retrieved from other groups of sauropsids, such as lizards, snakes, and tuatara. However, the latter are phylogenetically more distant to birds than turtles. The phylogenetic position of turtles (Testudines) in amniotes, and more precisely in sauropsids, has long been controversial, but phylogenomic studies indeed placed Testudines as a sister group of Archosauria [22,54].

### 3.7. Phylogeny of N- and C-Terminal Domains of AvBD11 among AvBDs and Turtle Defensins

**Figure 8:**
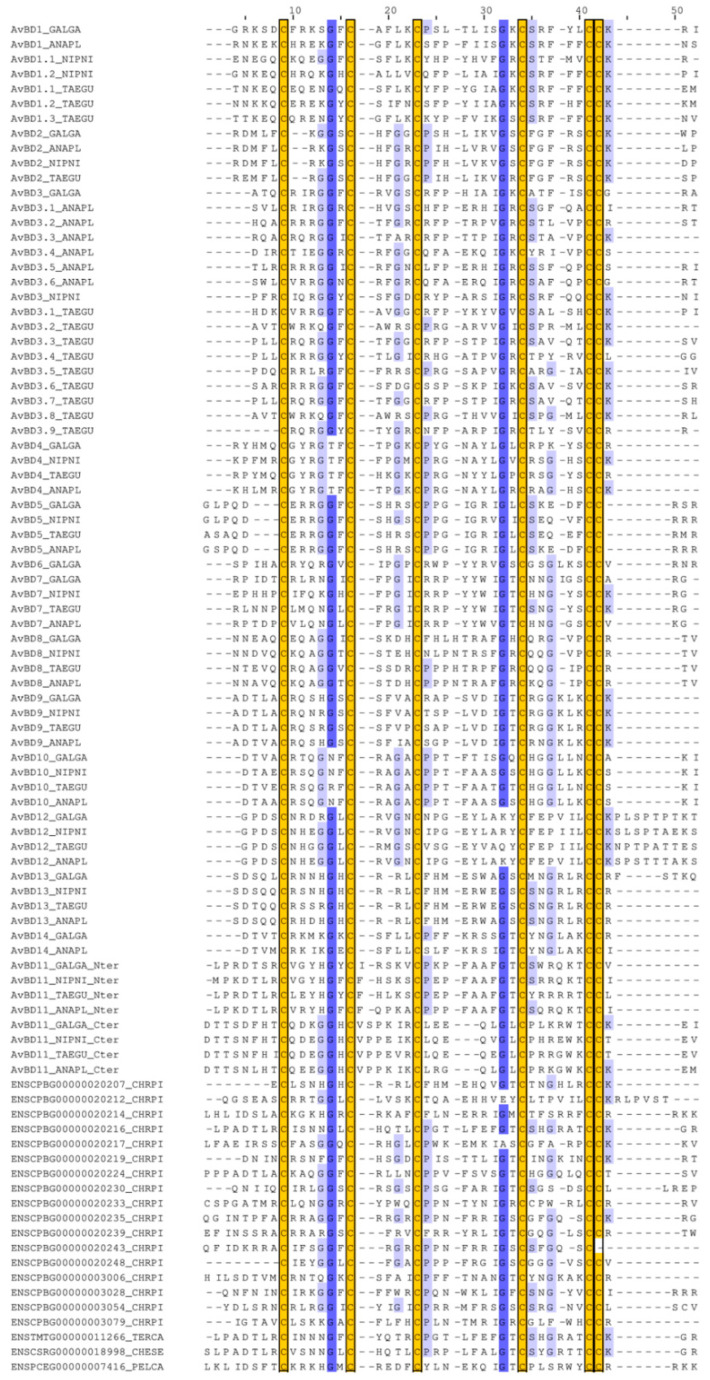
MSA of the N-terminal and C-terminal domains of AvBD11 with monodomain AvBDs and turtle β-defensins. MSA was performed with MAFFT and drawn with Jalview with a blue colour gradient (100% of identity for dark blue) and trimmed at each extremity to adjust to the length of the N-terminal and C-terminal domains of AvBD11. Conserved cysteine residues are highlighted in yellow boxes. Bird species acronyms: ANAPL, *Anas platyrhynchos* (duck); GALGA, *Gallus gallus* (chicken); NIPNI, *Nipponia nippon* (crested ibis); and TAEGU, *Taeniopygia guttata* (zebra finch). Turtle species acronyms: CHRPI, *Chrysemys picta bellii* (western painted turtle); TERCA, *Terrapene carolina triunguis* (three-toed box turtle); CHESE, *Chelydra serpentina* (common snapping turtle); and PELCA, *Pelusios castaneus* (west african mud turtle).

Following these findings, we investigated the phylogenetic relationships between avian β-defensins and putative turtle β-defensins, including a panel of defensins retrieved from the painted turtle *Chrysemys picta bellii* genome in Ensembl and those specifically identified in our BLAST analysis. A total of 17 putative β-defensin-coding genes were identified in the vicinity of CTSB and XKR5/TRAM2 genes in the painted turtle genome available in Ensembl. It is noteworthy that all of these putative defensins are monodomain. The MSA of monodomain AvBDs and turtle β-defensins used for the phylogenetic tree construction is presented in Figure 8.

**Figure 9:**
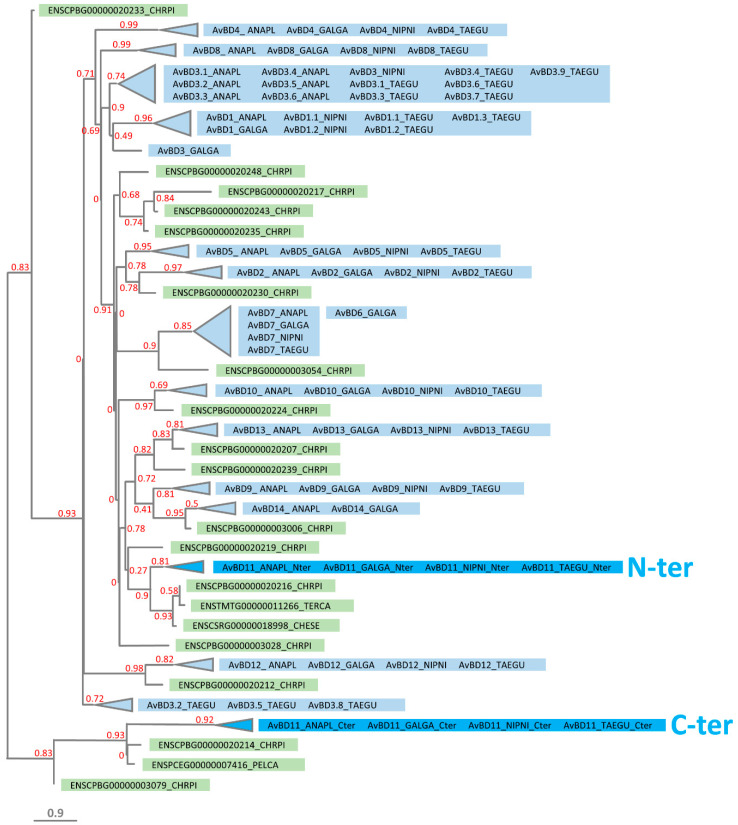
Phylogenetic relationships of the N-terminal (N-ter) and C-terminal (C-ter) domains of AvBD11 with monodomain AvBDs and turtle β-defensins. The phylogenetic tree was constructed using the maximum-likelihood-based program PhyML from MAFFT-based alignments adjusted to the length of N-ter and C-ter AvBD11. Branch support values are indicated in red. Bird and turtle β-defensins are highlighted in blue and in green, respectively. Bird species acronyms: ANAPL, *Anas platyrhynchos* (duck); GALGA, *Gallus gallus* (chicken); NIPNI, *Nipponia nippon* (crested ibis); and TAEGU, *Taeniopygia guttata* (zebra finch). Turtle species acronyms: CHRPI, *Chrysemys picta bellii* (western painted turtle); TERCA, *Terrapene carolina triunguis* (three-toed box turtle); CHESE, *Chelydra serpentina* (common snapping turtle); and PELCA, *Pelusios castaneus* (west african mud turtle).

Consistently with our BLAST results, the tree analysis (Figure 9) demonstrates that the C-terminal domain is associated with ENSPCEG00000007416 but also with ENSCPBG00000020214 and ENSCPBG00000003079 (0.83 branch support value), which is in accordance with the full MSA-based tree (Supplemental Data, Figure S6A) but not with the curated MSA-based tree (supplemental data, Figure S6B). In Figure 9, we also show that the N-terminal AvBD11 domain is closely associated to the three putative defensins identified by BLAST, namely ENSCPBG00000020216, ENSCSRG00000018998, and ENSTMTG00000011266 (0.9 branch support value). Results with the N-terminal domain are consistent with the tree obtained with the full and automatically curated MSAs (Supplemental Data, Figure S6). Similar to the previous results (crocodile β-defensins, Figure 6), our findings strongly support the hypothesis that the two domains of AvBD11: (i) are phylogenetically distant from each other, (ii) are more closely related with turtle defensins than with bird AvBDs, and (iii) result from two different ancestral genes, rather than from an internal duplication. To further appreciate the phylogenetic relationships of AvBD11 domains with turtle defensins, we performed new MSAs and tree analyses including crocodile and turtle β-defensins with either the N-terminal (Figures S10 and S11, Supplemental Data) or the C-terminal domain (Figures S12 and S13, Supplemental Data) of AvBD11. The tree in Figure S11 demonstrates that the N-terminal domain preferentially clusters with the previously identified turtle defensins (ENSCPBG00000020216, ENSCSRG00000018998, ENSTMTG00000011266) with 0.84 branch support value, but not with crocodile BD5. In contrast, in Figure S13, the C-terminal domain AvBD11 is isolated: no association is observed with turtle or crocodile defensins.

The tree analysis in Figure 9 also reveals that several AvBDs preferentially cluster with turtle defensins rather than AvBDs: AvBD2 and ENSCPG00000020230 (0.78 branch support value), AvBD7 and ENSCPG00000003054 (0.9 branch support value), AvBD10 and ENSCPG00000020224 (0.97 branch support value), AvBD12 and ENSCPG00000020212 (0.98 branch support value), AvBD13 and ENSCPG00000020207 (0.83 branch support value), AvBD14 and ENSCPG00000003006 (0.95 branch support value). Strikingly, the same group of AvBDs was previously found to preferentially associate with crocodile defensins (Figure 6). Our results strongly suggest that these defensins appeared before the divergence of Archelosauria.

Interestingly, the genomic alignment tool in Ensembl reveals that the two first exons of the chicken AvBD11 gene (encoding the signal peptide and the first defensin domain, respectively) are aligned with the two annotated exons of the painted turtle homolog gene ENSCPBG00000020216 (Supplemental Data, Figure S14). Very strikingly, although this latter gene is not annotated as a double-defensin in Ensembl, a genomic DNA sequence located in the downstream region of ENSCPBG00000020216 gene matches with the third exon of AvBD11 (corresponding to the C-terminal β-defensin domain; Figure S14) and virtually encodes for a defensin. If the exon annotation of ENSCPBG00000020216 is accurate, the presence of such a genomic sequence may constitute an evolutionary trace of an ancient double-defensin in the turtle genome. It is also possible that this region may correspond to an active exon, either fused with ENSCPBG00000020216 to produce a double-defensin, or forming an independent monodomain defensin. It is known that automated genome annotations in Ensembl can generate errors and lead to ‘missing genes’. Regardless of the situation, one could also hypothesize from these findings that an ancestral double-defensin might have arisen before the split between Testudines and Archosauria, thereafter evolving to AvBD11 in birds.

## 4. Conclusions

AvBD11 is a double-β-defensin composed of two β-defensin domains, which could carry different functions. Although the C-terminal domain has undergone important charge modifications during bird diversification, the evolution of AvBD11 is unlikely to have been driven by positive selection. The phylogenetic analyses revealed that the two domains are evolutionarily distant from OvoDs and distant from each other within the AvBDs. Quite importantly, some non-avian sauropsid defensins were even found phylogenetically closer to the AvBD11 domains, compared with any other AvBDs. Our findings demonstrate that the two domains of AvBD11 have common ancestors with different putative monodomain defensins of crocodile and turtle species, suggesting that AvBD11 may have arisen from the fusion of different ancestral monodomain defensins or possibly from an ancient double-defensin which originated before the Testudines-Archosauria split, rather than from a recent internal gene/exon duplication event. Our study also suggests that some AvBDs including AvBD2, AvBD7, AvBD10, AvBD12, AvBD13 and AvBD14 may have arisen before the divergence of Archelosauria and evolved independently in turtles, crocodiles and birds. The similarity of the N-terminal AvBD11 domain with turtle homologs, which is higher than with crocodile homologs, suggests that the AvBD11-related defensins may have rapidly evolved in crocodiles. However, the reason for such a degree of divergence in crocodile remains unknown. Although only monodomain defensins were reported in crocodile and turtle species by published studies to date, the absence of multidomain defensins in crocodile and turtle species should be carefully considered with regard to some recent data available in genome databases that may indicate the opposite. As an interesting perspective to this work, a similar approach could be applied to other multidomain defensins (such as those identified in lizard species) to better understand the evolutionary origin of the defensin repeated domains composing these ‘polydefensins’. Moreover, the exact molecular mechanisms and the underlying evolutionary forces leading to the appearance of such multidomain defensins during the evolution in specific clades will need to be further investigated.

## Data Availability

Not applicable.

## Supplementary Materials

The following supporting information can be downloaded at: https://www.mdpi.com/article/10.3390/biology11050690/s1, Figure S1: Full (untrimmed) MSA of monodomain AvBDs and OvoDs; Figure S2: Phylogenetic trees of monodomain AvBDs and OvoDs obtained from (A) full MSA (non-curated) and from (B) automatically curated MSA (removal of gap positions); Figure S3: Full (untrimmed) MSA of monodomain AvBDs and crocodile beta-defensins; Figure S4: Phylogenetic trees of monodomain AvBDs and crocodile beta-defensins obtained from (A) full MSA (non-curated) and from (B) automatically curated MSA (removal of gap positions); Figure S5: Full (untrimmed) MSA of monodomain AvBDs and turtle beta-defensins used for the construction of phylogenetic trees; Figure S6: Phylogenetic trees of monodomain AvBDs and turtle beta-defensins obtained from (A) full MSA (non-curated) and from (B) automatically curated MSA (removal of gap positions); Figure S7: Representation of the phylogenetic tree of bird AvBD11 sequences with the omega value (all are <1), showing that there is no branch site positive selection; Figure S8: MSA of mature AvBD11 protein sequences in various bird species showing examples of amino acid positions under potential convergent evolution; Figure S9: Backbone of Gga-AvBD11 (6QEU.pdb) rainbow colored from N-ter (in blue) to C-ter (in red) with disulfide bridges in sticks. Sidechains of W33 and R75 are represented in brown and blue, respectively; Figure S10: MSA of the N-terminal domain of AvBD11 with monodomain crocodile and turtle β-defensins; Figure S11: Phylogenetic tree of the N-terminal domain of AvBD11 with monodomain crocodile and turtle β-defensins; Figure S12: MSA of the C-terminal domain of AvBD11 with monodomain crocodile and turtle β-defensins; Figure S13: Phylogenetic tree of the C-terminal domain of AvBD11 with monodomain crocodile and turtle β-defensins; Figure S14: Genomic alignment of chicken AvBD11 gene and painted turtle ENSCPBG00000020216 gene; Table S1: List of acronyms used for animal species used in the present study; Table S2: List of protein sequences used for the construction of multiple sequence alignments (MSAs) and phylogenetic trees; Table S3: List of nucleotide sequences used for the positive selection analysis.

## References

[1] Shafee T.M., Lay F.T., Phan T.K., Anderson M.A., Hulett M.D. (2017). Convergent evolution of defensin sequence, structure and function. Cell Mol. Life Sci..

[2] Shafee T.M., Lay F.T., Hulett M.D., Anderson M.A. (2016). The Defensins Consist of Two Independent, Convergent Protein Superfamilies. Mol. Biol. Evol..

[3] Lehrer R.I., Lu W. (2012). alpha-Defensins in human innate immunity. Immunol. Rev..

[4] Hughes A.L. (1999). Evolutionary diversification of the mammalian defensins. Cell Mol. Life Sci..

[5] Semple C.A., Taylor K., Eastwood H., Barran P.E., Dorin J.R. (2006). Beta-defensin evolution: Selection complexity and clues for residues of functional importance. Biochem. Soc. Trans..

[6] Cheng Y., Prickett M.D., Gutowska W., Kuo R., Belov K., Burt D.W. (2015). Evolution of the avian beta-defensin and cathelicidin genes. BMC Evol. Biol..

[7] Zhang L., Chen D., Yu L., Wei Y., Li J., Zhou C. (2019). Genome-wide analysis of the ovodefensin gene family: Monophyletic origin, independent gene duplication and presence of different selection patterns. Infect Genet Evol..

[8] Whenham N., Lu T.C., Maidin M.B., Wilson P.W., Bain M.M., Stevenson M.L., Stevens M.P., Bedford M.R., Dunn I.C. (2015). Ovodefensins, an Oviduct-Specific Antimicrobial Gene Family, Have Evolved in Birds and Reptiles to Protect the Egg by Both Sequence and Intra-Six-Cysteine Sequence Motif Spacing. Biol. Reprod.

[9] Zhang G., Sunkara L.T. (2014). Avian antimicrobial host defense peptides: From biology to therapeutic applications. Pharmaceuticals.

[10] Guyot N., Meudal H., Trapp S., Iochmann S., Silvestre A., Jousset G., Labas V., Reverdiau P., Loth K., Herve V. (2020). Structure, function, and evolution of Gga-AvBD11, the archetype of the structural avian-double-beta-defensin family. Proc. Natl. Acad. Sci. USA.

[11] Herve V., Meudal H., Labas V., Rehault-Godbert S., Gautron J., Berges M., Guyot N., Delmas A.F., Nys Y., Landon C. (2014). Three-dimensional NMR structure of Hen Egg Gallin (Chicken Ovodefensin) reveals a new variation of the beta-defensin fold. J. Biol. Chem..

[12] Mann K. (2008). Proteomic analysis of the chicken egg vitelline membrane. Proteomics.

[13] Kido S., Morimoto A., Kim F., Doi Y. (1992). Isolation of a novel protein from the outer layer of the vitelline membrane. Biochem. J..

[14] Mann K. (2007). The chicken egg white proteome. Proteomics.

[15] Lim W., Jeong W., Kim J., Yoshimura Y., Bazer F.W., Han J.Y., Song G. (2013). Expression and regulation of beta-defensin 11 in the oviduct in response to estrogen and in ovarian tumors of chickens. Mol. Cell Endocrinol..

[16] Herve-Grepinet V., Rehault-Godbert S., Labas V., Magallon T., Derache C., Lavergne M., Gautron J., Lalmanach A.C., Nys Y. (2010). Purification and characterization of avian beta-defensin 11, an antimicrobial peptide of the hen egg. Antimicrob Agents Chemother.

[17] Dalla Valle L., Benato F., Maistro S., Quinzani S., Alibardi L. (2012). Bioinformatic and molecular characterization of beta-defensins-like peptides isolated from the green lizard Anolis carolinensis. Dev. Comp. Immunol..

[18] Fry B.G., Roelants K., Winter K., Hodgson W.C., Griesman L., Kwok H.F., Scanlon D., Karas J., Shaw C., Wong L. (2010). Novel venom proteins produced by differential domain-expression strategies in beaded lizards and gila monsters (genus *Heloderma*). Mol. Biol. Evol..

[19] van Hoek M.L., Prickett M.D., Settlage R.E., Kang L., Michalak P., Vliet K.A., Bishop B.M. (2019). The Komodo dragon (Varanus komodoensis) genome and identification of innate immunity genes and clusters. BMC Genom..

[20] Santana F.L., Estrada K., Ortiz E., Corzo G. (2021). Reptilian beta-defensins: Expanding the repertoire of known crocodylian peptides. Peptides.

[21] Yu H., Wang H., Liu X., Feng L., Qiao X., Cai S., Shi N., Wang Y. (2017). Identification, eukaryotic expression and structure & function characterizations of beta-defensin like homologues from Pelodiscus sinensis. Dev. Comp. Immunol..

[22] Crawford N.G., Faircloth B.C., McCormack J.E., Brumfield R.T., Winker K., Glenn T.C. (2012). More than 1000 ultraconserved elements provide evidence that turtles are the sister group of archosaurs. Biol. Lett..

[23] Gemmell N.J., Rutherford K., Prost S., Tollis M., Winter D., Macey J.R., Adelson D.L., Suh A., Bertozzi T., Grau J.H. (2020). The tuatara genome reveals ancient features of amniote evolution. Nature.

[24] Waterhouse A.M., Procter J.B., Martin D.M., Clamp M., Barton G.J. (2009). Jalview Version 2--a multiple sequence alignment editor and analysis workbench. Bioinformatics.

[25] Katoh K., Standley D.M. (2013). MAFFT multiple sequence alignment software version 7: Improvements in performance and usability. Mol. Biol. Evol..

[26] Dereeper A., Guignon V., Blanc G., Audic S., Buffet S., Chevenet F., Dufayard J.F., Guindon S., Lefort V., Lescot M. (2008). Phylogeny.fr: Robust phylogenetic analysis for the non-specialist. Nucleic Acids Res..

[27] Guindon S., Gascuel O. (2003). A simple, fast, and accurate algorithm to estimate large phylogenies by maximum likelihood. Syst. Biol..

[28] Anisimova M., Gil M., Dufayard J.F., Dessimoz C., Gascuel O. (2011). Survey of branch support methods demonstrates accuracy, power, and robustness of fast likelihood-based approximation schemes. Syst. Biol..

[29] Meslin C., Mugnier S., Callebaut I., Laurin M., Pascal G., Poupon A., Goudet G., Monget P. (2012). Evolution of genes involved in gamete interaction: Evidence for positive selection, duplications and losses in vertebrates. PLoS ONE.

[30] Grandchamp A., Monget P. (2020). The membrane receptors that appeared before their ligand: The different proposed scenarios. PLoS ONE.

[31] Yang Z. (2007). PAML 4: Phylogenetic analysis by maximum likelihood. Mol. Biol. Evol..

[32] Storey J.D., Tibshirani R. (2003). Statistical significance for genomewide studies. Proc. Natl. Acad. Sci. USA.

[33] Shultz A.J., Sackton T.B. (2019). Immune genes are hotspots of shared positive selection across birds and mammals. Elife.

[34] Meslin C., Brimau F., Nagnan-Le Meillour P., Callebaut I., Pascal G., Monget P. (2011). The evolutionary history of the SAL1 gene family in eutherian mammals. BMC Evol. Biol..

[35] Dufourny L., Levasseur A., Migaud M., Callebaut I., Pontarotti P., Malpaux B., Monget P. (2008). GPR50 is the mammalian ortholog of Mel1c: Evidence of rapid evolution in mammals. BMC Evol. Biol..

[36] Roy A., Kucukural A., Zhang Y. (2010). I-TASSER: A unified platform for automated protein structure and function prediction. Nat. Protoc..

[37] Rivas E., Eddy S.R. (2008). Probabilistic phylogenetic inference with insertions and deletions. PLoS Comput. Biol..

[38] Saurabh K., Holland B.R., Gibb G.C., Penny D. (2012). Gaps: An elusive source of phylogenetic information. Syst. Biol..

[39] Donath A., Stadler P.F. (2018). Split-inducing indels in phylogenomic analysis. Algorithms Mol. Biol..

[40] Dessimoz C., Gil M. (2010). Phylogenetic assessment of alignments reveals neglected tree signal in gaps. Genome Biol..

[41] Meade K.G., Higgs R., Lloyd A.T., Giles S., O’Farrelly C. (2009). Differential antimicrobial peptide gene expression patterns during early chicken embryological development. Dev. Comp. Immunol..

[42] Hellgren O., Ekblom R. (2010). Evolution of a cluster of innate immune genes (beta-defensins) along the ancestral lines of chicken and zebra finch. Immunome Res..

[43] Gilroy D., van Oosterhout C., Komdeur J., Richardson D.S. (2016). Avian β-defensin variation in bottlenecked populations: The Seychelles warbler and other congeners. Conserv. Genet..

[44] Lu S., Peng K., Gao Q., Xiang M., Liu H., Song H., Yang K., Huang H., Xiao K. (2014). Molecular cloning, characterization and tissue distribution of two ostrich beta-defensins: AvBD2 and AvBD7. Gene.

[45] Chen H., Ma M.Y., Sun L., Fang S.G., Wan Q.H. (2015). Genomic structure and evolution of beta-defensin genes in the golden pheasant and hwamei. Sci. Bull..

[46] Lan H., Chen H., Chen L.C., Wang B.B., Sun L., Ma M.Y., Fang S.G., Wan Q.H. (2014). The first report of a Pelecaniformes defensin cluster: Characterization of beta-defensin genes in the crested ibis based on BAC libraries. Sci. Rep..

[47] Ishige T., Hara H., Hirano T., Mannen H., Kono T., Hanzawa K. (2016). Basic characterization of avian beta-defensin genes in the Japanese quail, Coturnix japonica. Anim. Sci. J..

[48] Ma D., Lin L., Zhang K., Han Z., Shao Y., Wang R., Liu S. (2012). Discovery and characterization of Coturnix chinensis avian beta-defensin 10, with broad antibacterial activity. J. Pept. Sci..

[49] Hellgren O., Sheldon B.C. (2011). Locus-specific protocol for nine different innate immune genes (antimicrobial peptides: Beta-defensins) across passerine bird species reveals within-species coding variation and a case of trans-species polymorphisms. Mol. Ecol. Resour..

[50] Lynn D.J., Higgs R., Lloyd A.T., O’Farrelly C., Herve-Grepinet V., Nys Y., Brinkman F.S., Yu P.L., Soulier A., Kaiser P. (2007). Avian beta-defensin nomenclature: A community proposed update. Immunol. Lett..

[51] Zhou C., Wang G., Yu H., Geng Y., Wu W., Tu H., Price M., Fan Z., Meng Y., Yue B. (2019). Genome-wide analysis reveals the genomic features of the turkey vulture (Cathartes aura) as a scavenger. Mol. Genet. Genom..

[52] Xiao Y., Hughes A.L., Ando J., Matsuda Y., Cheng J.F., Skinner-Noble D., Zhang G. (2004). A genome-wide screen identifies a single beta-defensin gene cluster in the chicken: Implications for the origin and evolution of mammalian defensins. BMC Genom..

[53] Pais F.S., Ruy P.C., Oliveira G., Coimbra R.S. (2014). Assessing the efficiency of multiple sequence alignment programs. Algorithms Mol. Biol..

[54] Chiari Y., Cahais V., Galtier N., Delsuc F. (2012). Phylogenomic analyses support the position of turtles as the sister group of birds and crocodiles (Archosauria). BMC Biol..

