# Supplementary material for: The Two Domains of the Avian Double-β-Defensin AvBD11 Have Different Ancestors, Common with Potential Monodomain Crocodile and Turtle Defensins
Source: Biology (Basel). 2022 Apr 30;11(5):690. doi: 10.3390/biology11050690 (PMC9138766; doi:10.3390/biology11050690)

Figure S12. MSA of the C-terminal domain of AvBD11 with monodomain crocodile and turtle β-defensins. MSA was performed with MAFFT (BLOSUM40 matrix, gap penalty 1.7, manual adjustments) and drawn with Jalview with a blue colour gradient (100% of identity for dark blue) and trimmed at each extremity to adjust to the length of the C-terminal domain of AvBD11. Conserved cysteine residues are highlighted in yellow boxes. Bird species acronyms: ANAPL, *Anas plathyrhynchos* (duck); GALGA, *Gallus gallus* (chicken); NIPNI, *Nipponia nippon* (crested ibis); and TAEGU, *Taeniopygia guttata* (zebra finch). Crocodile species acronyms: ALLMI, *Alligator mississippiensis* (American alligator); ALLSI, *Alligator sinensis* (Chinese alligator); CROPO, *Crocodylus porosus* (saltwater crocodile); and GAVGA, *Gavialis gangeticus* (gharial). Turtle species acronyms: CHRPI, *Chrysemys picta bellii* (western painted turtle); TERCA, *Terrapene carolina triunguis* (three-toed box turtle); CHESE, *Chelydra serpentina* (common snapping turtle); and PELCA, *Pelusios castaneus* (west african mud turtle).

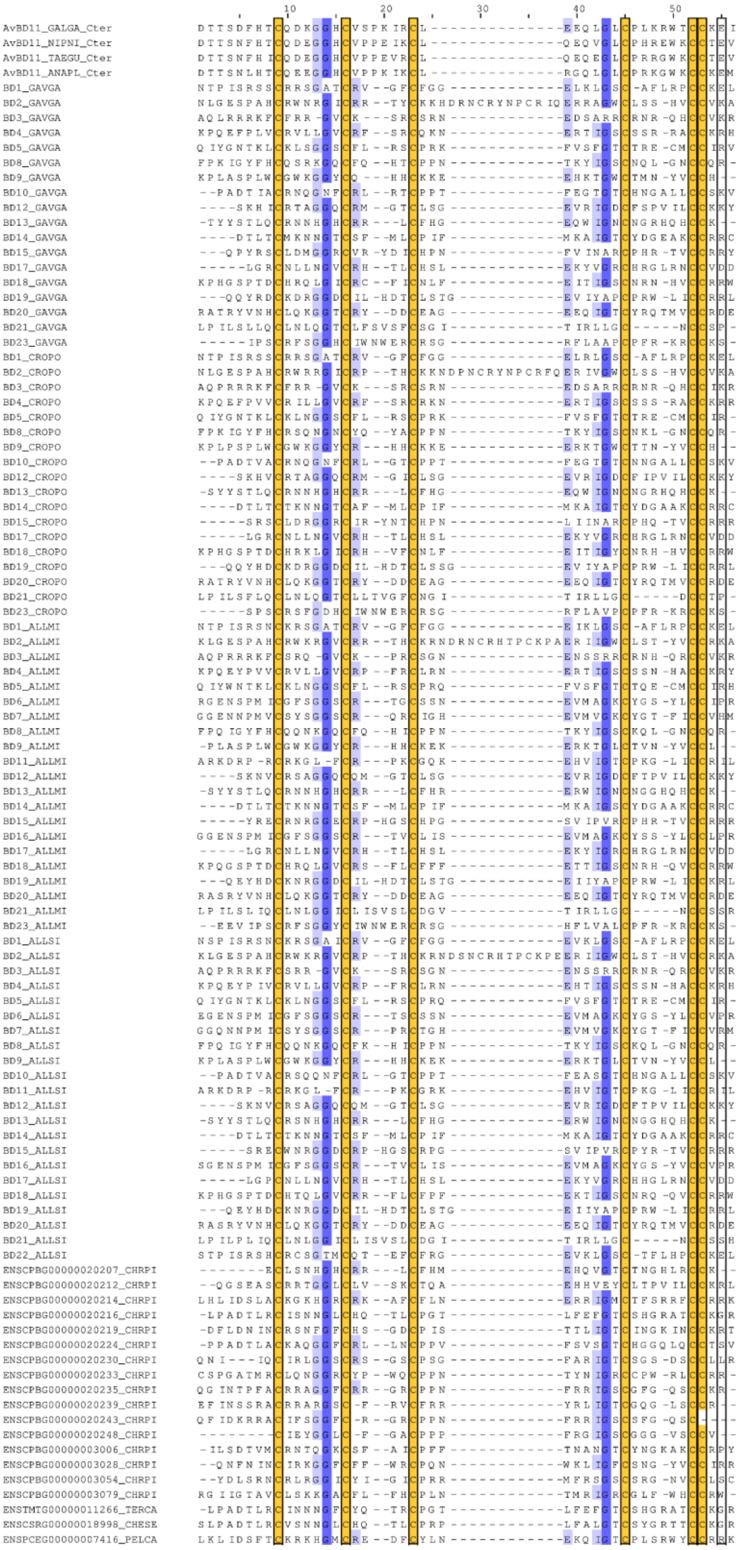

Supplement: Supplementary file 1 [file biology-11-00690-s001.zip › Figure S12.pdf]
