# Supplementary material for: The Two Domains of the Avian Double-β-Defensin AvBD11 Have Different Ancestors, Common with Potential Monodomain Crocodile and Turtle Defensins
Source: Biology (Basel). 2022 Apr 30;11(5):690. doi: 10.3390/biology11050690 (PMC9138766; doi:10.3390/biology11050690)
Supplement: Supplementary file 1 [file biology-11-00690-s001.zip › Figure S14.pdf]

Figure S14. Genomic alignment of chicken AvBD11 gene and painted turtle ENSCPBG00000020216 gene. This genomic alignment has been imported from Ensembl (www.ensembl.org) and further formatted to identify exons, start/stop codons and protein-coding regions. Exons identified in Ensembl database are highlighted in blue (exon 1), in green (exon 2) and in yellow (exon 3). Start and stop codons are highlighted in red and black, respectively. Protein-coding regions are represented in bold. Conserved nucleotides are represented with an asterisk. Note that the signal peptide, the N-terminal β-defensin domain and the C-terminal β-defensin domain of chicken AvBD11 are encoded by exons 1, 2 and 3, respectively.

Chicken › chromosome:GRCg6a:3:107405631:107409449:-1

Painted turtle › primary_assembly:Chrysemys_picta_bellii-3.0.3:KK083003.1:3221761:3222172:-1
primary_assembly:Chrysemys_picta_bellii-3.0.3:KK083003.1:3219393:3219698:-1
primary_assembly:Chrysemys_picta_bellii-3.0.3:KK083003.1:3217671:3218026:-1

```
CLUSTAL W (1.81) multiple sequence alignment

gallus_gallus/1-2640          CTACCATGAAGCTCTTCTCCTGCCTCATGGCTCTGCTCCTCTTCCTCCTCCAGGCTGTTC
chrysemys_picta_bellii/1-2640 CCACCATGAAGATCCTTTACCTGATCTTTGCTGTTCTCCTCTTCCTCCTCCAGGCAACTC
                              * ********* ** * * *    ** * *** * ********************   **

gallus_gallus/1-2640          CAGGTAAGGAGCAACACCGGTGCTGGGGTCTGCCTGTAGGAAGCAGTGAATCTTGTTGCG
chrysemys_picta_bellii/1-2640 CAGGTAAGGCGTAA-ACCAGAGGAGAGGGATGATTGTGGG------------TAATCGCA
                              ********* * ** *** * *  * **  **  *** **            *  * ** 

gallus_gallus/1-2640          TAGGGGGCACAAGTTGTGTCATGGTGCAGACTGTATTTCCCTGTGCTGATCAGCAACTTT
chrysemys_picta_bellii/1-2640 TGAGGAG---ATGTTACAACA--GTGCGGACAACATATTTCACTGCTTTTCAGTTCCTTA
                              *  ** *   * ***    **  **** ***   ** *  *  ****  ****   *** 

gallus_gallus/1-2640          CGTTAGAGGTG------ATTCCTTAGTCCAGAAACTGAAGTGAAAAATGATCCCCAAGTA
chrysemys_picta_bellii/1-2640 CATTAAAGCTGTTCCCTGTTTCTCAGTCCAGA............................
                              * *** ** **       ** ** ********

gallus_gallus/1-2640          ACCAGTCATCAAAGCAGAGCACTCCTAGAGATGCCACTGCTGCTGGCATGGTGTGTCACC
chrysemys_picta_bellii/1-2640 ............................................................

gallus_gallus/1-2640          ATGGTACGCTGGTCTCCTTCAAACTCTGTGAAGTTTCTTTATGACGTTTTGTGGCACTGC
chrysemys_picta_bellii/1-2640 ............................................................

gallus_gallus/1-2640          TGGAAGAAGGCACAAAGGAAATGCTTCTTCATAACCCAAGGTCACCAGTCTTCCCACTGG
chrysemys_picta_bellii/1-2640 ............................................................

gallus_gallus/1-2640          GGAACTGTAGCAGAGGAGTAGAGGTACCCCCTTGCATTACCCAAGTTGAACTACCCCAGT
chrysemys_picta_bellii/1-2640 ............................................................

gallus_gallus/1-2640          GAAGTTTACCTAAGTTCTCTGAATATGCTGACACCCCTTCCCCAGACCTTCAGTTGCATT
chrysemys_picta_bellii/1-2640 ............................................................

gallus_gallus/1-2640          CATTTACTGTTTGTGCTTTTAGCCTAACTTTTCTTCATGCCTCATGAAACTCCCCATCCT
chrysemys_picta_bellii/1-2640 ............................................................

gallus_gallus/1-2640          AGGGTAAGGCTTTCAAAAGGGAAGAAATTGGATAAATATCTTCTCAGCAATAACTGGACC
chrysemys_picta_bellii/1-2640 ............................................................

gallus_gallus/1-2640          AGATCCCAACATTTAATTTTGGTTAAGTTATAGGTACAGCTCTGGGCATGCAAGCACATT
chrysemys_picta_bellii/1-2640 ............................................................

gallus_gallus/1-2640          GCTCTCCCTTCCATGACCAGCCTGTAGCACTGCAGTCCTGGAACTAACAACTACCCCTTA
chrysemys_picta_bellii/1-2640 ............................................................

gallus_gallus/1-2640          TGATGTGCATGATGTGCAGTGAGTAGGATATGTTCAGAAACTCAGCAGGTGTTGTTTTAC
chrysemys_picta_bellii/1-2640 ............................................................

gallus_gallus/1-2640          TCCAGTTGTACAAAGTCCTTCTTTGGTGTCCCGTGAGGTCCATGAGCATACTCAAAACCT
```

```
chrysemys_picta_bellii/1-2640 ............................................................

gallus_gallus/1-2640          TCAGACAAACTGTCTTGTGCTTTGGGGGTCTTTGTGTCACACCCCTAATAGGGGTCCACG
chrysemys_picta_bellii/1-2640 ............................................................

gallus_gallus/1-2640          GGGTCTCAGCAGGAGGAAGATCGTGAGACATGGACTGATCCTGCAGCACAACTGGGAATC
chrysemys_picta_bellii/1-2640 ...............................GGGCCGATATCGCAGCACTTCTCAGACTC
                                                             ** * ***   *******  **  ** **

gallus_gallus/1-2640          CC-ATTCTTCCTGTAGGTCTCGGCTTGCCCAGAGACACCTCACGTTGTGTTGGCTACCAT
chrysemys_picta_bellii/1-2640 CCCGTTCCCCCTGCAGGGCTCAGCCTGCCTGCAGACACCCTGAGATGTATCAGCAACAAT
                              **  ***  **** *** *** ** ****   *******    * *** *  ** ** **

gallus_gallus/1-2640          GGGTACTGCATCCGTTCCAAAGTCTGCCCCAAACCATTTGCTGCATTTGGAACATGCTCT
chrysemys_picta_bellii/1-2640 GGCCTCTGCCACCA---AACACTCTGCCCTGGAACACTGTTCGAATTTGGAACCTGCTCG
                              **   ****  **     * * *******   * ** *    * ********* *****

gallus_gallus/1-2640          TGGCGTCAGAAAACCTGCTGCGTAGGTATGT-GTGGGAACCCTGCCCAG-GCAGGCAATG
chrysemys_picta_bellii/1-2640 CACGGTCGTGCGACCTGCTGTAAAGGTAGGTGGTGAGGATCCTGCTTGGAGCTGATGCAA
                                  ***     ********   ***** ** *** * * *****   * ** *

gallus_gallus/1-2640          TCTGGAGAGGAGAATACTGAAG--TCAGCAAAACGATCTCTTTATCTC---ATCTCCAAA
chrysemys_picta_bellii/1-2640 TCTGCAG-GGGAACATTGTAGCATCAGGAGACAAATTTGTTGCTCTCTTTGTCTCCAAA
                              **** ** ** *** * ** **  **** * *   ** * **  ****    ********

gallus_gallus/1-2640          CAGTCCAAGACCTCTCCTTTCGTCTACACAGATGGATCTGGCAGTCCCTGTGCTAATGGC
chrysemys_picta_bellii/1-2640 CTTCTTAAATCCTCCTTCTTTGT-GAAAAAGATAGATCTTGC..................
                              *     **  ****    ** **  * * **** ***** **

gallus_gallus/1-2640          AGCACGGAGCCCAAGAACATAGCTGCCATGTCACGTTAGCATAAAGCCCAGTGTTCTGTT
chrysemys_picta_bellii/1-2640 ............................................................

gallus_gallus/1-2640          TCCTACAGAGCAGCAGTTTGGTGAAAGAACACAAAAATTGTGGCCCCCATAGAGTATGCT
chrysemys_picta_bellii/1-2640 ............................................................

gallus_gallus/1-2640          TTCATTGATGTATATTCCCAGTATCCTCTGTCTACTGGAGGACTGCTGGGTGCATCCACG
chrysemys_picta_bellii/1-2640 ............................................................

gallus_gallus/1-2640          ATGATATGTTTAACAGGCACCAGTTGACTTCCTTCCTGTAAACCCATCCAGTCCTTTATG
chrysemys_picta_bellii/1-2640 ............................................................

gallus_gallus/1-2640          GACCTGCTTCCTTTTGGAGCTCCTCAACAATCAATTGATGTCTTTATATCCATACATGCT
chrysemys_picta_bellii/1-2640 ............................................................

gallus_gallus/1-2640          TAATAGCCAGGTGTGCTAATCTGACCCAGAGTTCATCCAGCACCCTGTATCTGACCTCAG
chrysemys_picta_bellii/1-2640 ............................................................

gallus_gallus/1-2640          CCAGAAGCAGATGCTTCTACACAGTCAGTCTGTGTGTGTGAAATGGATATCATGAACTCC
chrysemys_picta_bellii/1-2640 ............................................................

gallus_gallus/1-2640          ACAACAATATAATTACAATTTCTCAGATATTCTCTTGGCTTGCATGAATTGGCAGCTTTT
chrysemys_picta_bellii/1-2640 ............................................................

gallus_gallus/1-2640          AGACCTAGACATCCTTGTGTTTAAAAGTCCTCACTGGATTTTTTCCCCCCTGAAATTGGG
chrysemys_picta_bellii/1-2640 ............................................................

gallus_gallus/1-2640          TACATCTTCCCTATGTTTGAAAACCACTTCTCTCATTACCTAATTCTGCGTTGCAGTTGA
chrysemys_picta_bellii/1-2640 ............................................................

gallus_gallus/1-2640          AACGAATAGTCCTTGTGCAGCCATCTTCATAACTTCATCATACCCCCTCCTTCAGTTTCC
chrysemys_picta_bellii/1-2640 ............................................................

gallus_gallus/1-2640          CCTTCAAGGTGATATATCACAAACTCCTCCTCTTACAGAAGTGCTCTGAATATCCCCACA
chrysemys_picta_bellii/1-2640 ............................................................
```

```
gallus_gallus/1-2640          GTCCTTCTTTATGCCTTTGCTTTTGAGGACTACGAACCAGAACTTCCAATACTCTCTGTA
chrysemys_picta_bellii/1-2640 ............................................................


gallus_gallus/1-2640          TAGGTGTGCAGCAGTGAGTCAGATGGTGGTGTAATTATGTTTTGTGTTTTAACTTCAATC
chrysemys_picta_bellii/1-2640 ............................................................


gallus_gallus/1-2640          CTTCCCATTCCAGACACTACATCAGACTTCCATACTTGTCAAGACAAGGGGGGCCATTGC
chrysemys_picta_bellii/1-2640 ..........CAGGTATTTCTCCAGGCTCCCCTTCCTGTGCAGATAATGGGGGTGACTGC
                                        ***  * * *  *** ** ** * * ***  *** ** *****  * ***

gallus_gallus/1-2640          GTATCTCCAAAAATCAGATGTCTGGAAGAACAGCTGGGACTTTGCCCTCTAAAAAGATGG
chrysemys_picta_bellii/1-2640 ATACCCCCGGACGCAGACTGTCCCCAAGACCTCTCTGGAGCCTGCTCGCGCGGAATGTGG
                               ** * **  *       ****   **** *     ***   *** * *    **  ***

gallus_gallus/1-2640          ACGTGCTGCAAAGAAATATAATGAAGACCTTGCAGAATTGCAGAAAGCC-ACAGAAGTGC
chrysemys_picta_bellii/1-2640 AAATGCTGTAAACTAATCCCATAAAGATCTCACAGCACTGCACAAATCCAAAAGGGATGC
                              *  ***** ***  ***   ** **** **  *** * **** *** ** * **   ***

gallus_gallus/1-2640          ------ACAGTCAAGAAAACAAACAAGCTTTCTTGCTAGAGTTGTAGAGAAGCTGCCAGA
chrysemys_picta_bellii/1-2640 CTGAAAACATTAGGAAATGCATGTTACTTTCCTTTTCA----TATAGAGTAGCTGTTTAA
                                    *** *    **  **    *  ** ***   *    * ***** *****    *

gallus_gallus/1-2640          CTTTCTCAACATACGAAGTCTGCAATTCGTTAGAGGCGACGTGCTTTTTTAATCCACACA
chrysemys_picta_bellii/1-2640 CTGTCT-----TATTAAAAATATGATTTATCTGAAGAGACATGCGTCTCC-ATCTGTAAA
                              ** ***     **  **   *   ***  *  ** * *** *** * *   ***   * *

gallus_gallus/1-2640          ACAAAGGCAATAAACATTAAATAAACAGTGCAACAGCCCCATATATACACAGAGAGAGGC
chrysemys_picta_bellii/1-2640 ATTAAAACAATTAATACTAAATAAAGCAAAAAAAAATCAAATATTTAAATAAAGGGAGAA
                              *  **  **** ** * ********      ** *  *  **** ** * * ** ***

gallus_gallus/1-2640          AGAAGGTCTCTGTCTCACACACGCACATGTAGAAATGCACAGAAACACAGCTGTTAAACC
chrysemys_picta_bellii/1-2640 AGTGAATTTCTCTCTC............................................
                              **    * *** ****

gallus_gallus/1-2640          CTTGGAAACCACATCCCCTCCCACTCCAAACTTCACCTCCCTCTTCTTTCTGTCCCATCG
chrysemys_picta_bellii/1-2640 ............................................................
```
