# Supplementary material for: The Two Domains of the Avian Double-β-Defensin AvBD11 Have Different Ancestors, Common with Potential Monodomain Crocodile and Turtle Defensins
Source: Biology (Basel). 2022 Apr 30;11(5):690. doi: 10.3390/biology11050690 (PMC9138766; doi:10.3390/biology11050690)

Figure S2. Phylogenetic trees of monodomain AvBDs and OvoDs obtained from (A) full MSA (non-curated) and from (B) automatically curated MSA (removal of gap positions)

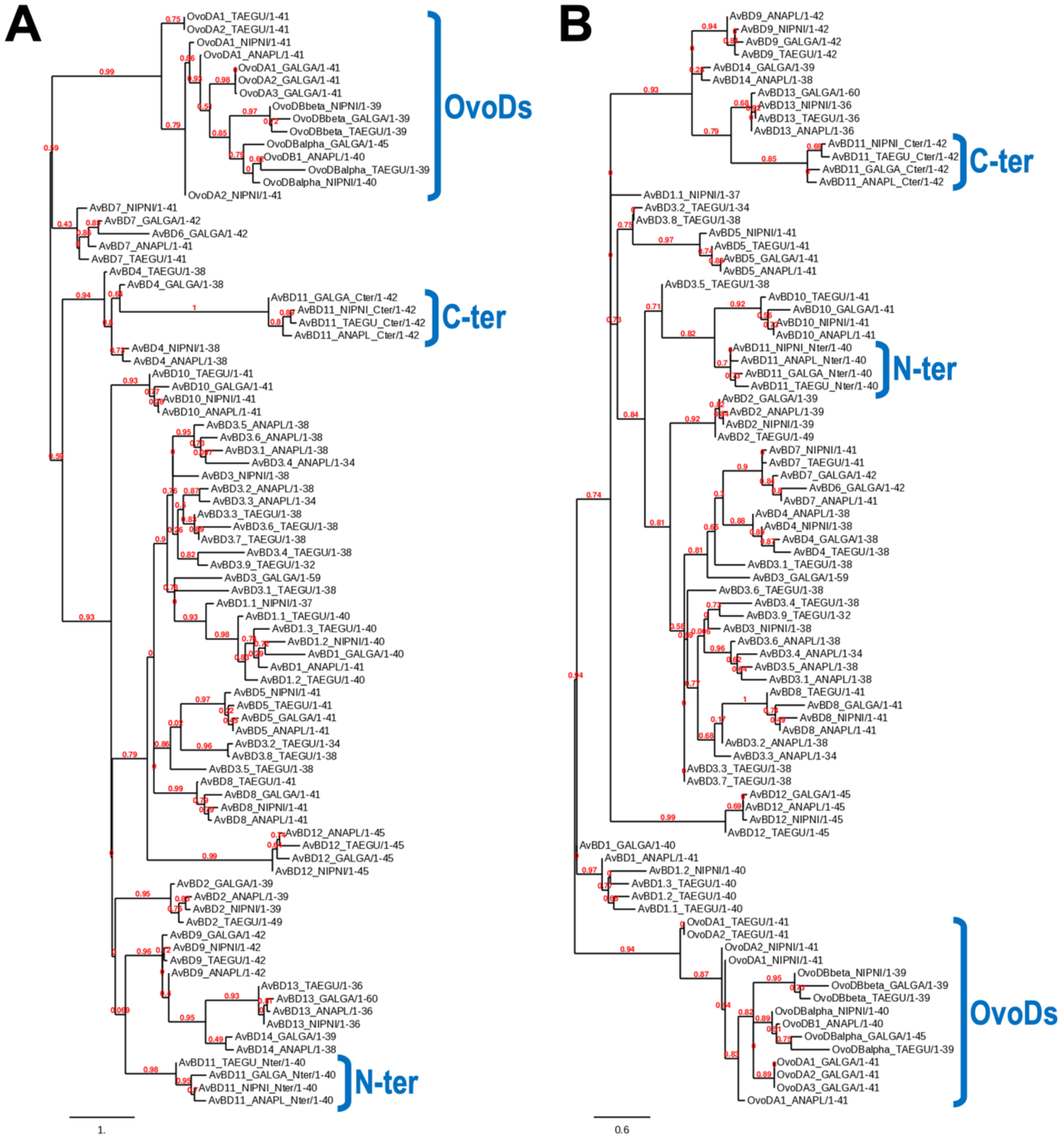

Supplement: Supplementary file 1 [file biology-11-00690-s001.zip › Figure S2.pdf]
