# Supplementary material for: The Two Domains of the Avian Double-β-Defensin AvBD11 Have Different Ancestors, Common with Potential Monodomain Crocodile and Turtle Defensins
Source: Biology (Basel). 2022 Apr 30;11(5):690. doi: 10.3390/biology11050690 (PMC9138766; doi:10.3390/biology11050690)

Figure S7. Representation of the phylogenetic tree of bird AvBD11 sequences with the omega value (all are < 1), showing that there is no branch site positive selection.

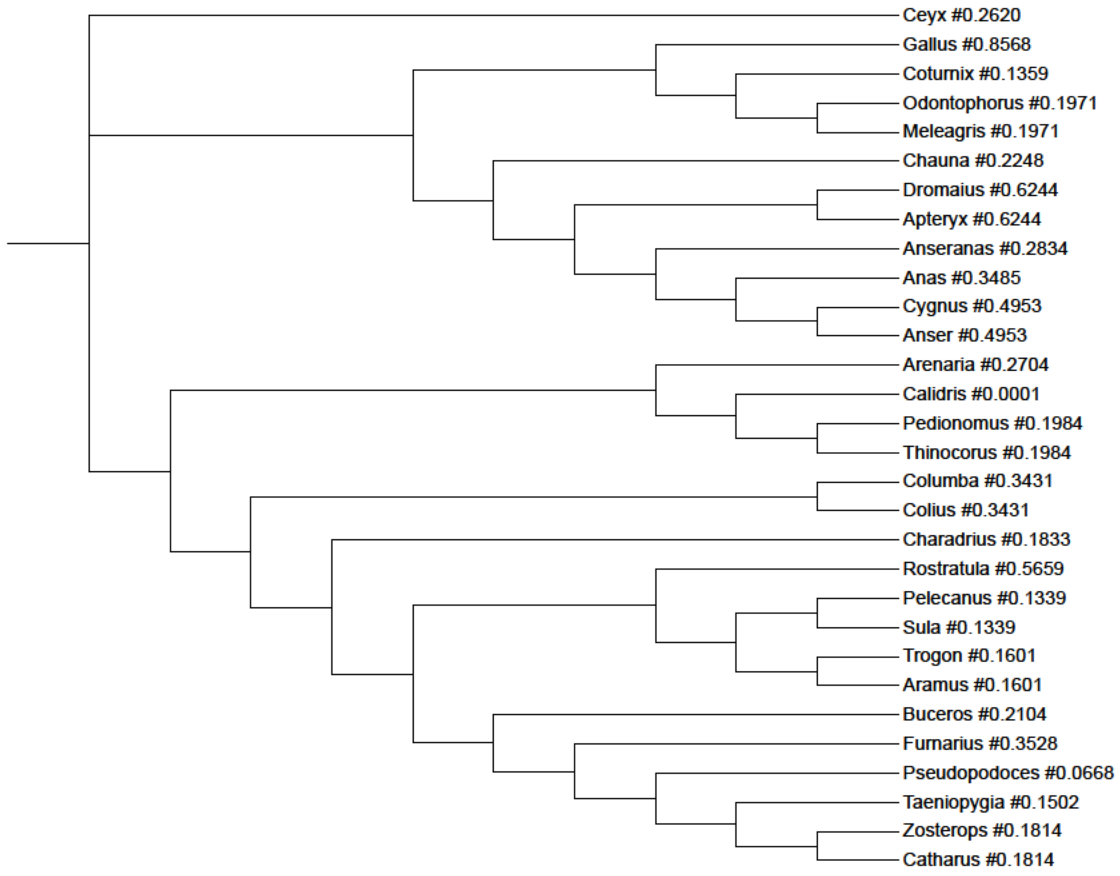

Supplement: Supplementary file 1 [file biology-11-00690-s001.zip › Figure S7.pdf]
