# Supplementary material for: The Two Domains of the Avian Double-β-Defensin AvBD11 Have Different Ancestors, Common with Potential Monodomain Crocodile and Turtle Defensins
Source: Biology (Basel). 2022 Apr 30;11(5):690. doi: 10.3390/biology11050690 (PMC9138766; doi:10.3390/biology11050690)

Figure S8. MSA of mature AvBD11 protein sequences in various bird species showing examples of amino acid positions under potential convergent evolution. All protein sequences were from our previous study (Guyot, N.; Meudal, H.; Trapp, S.; Iochmann, S.; Silvestre, A.; Jousset, G.; Labas, V.; Reverdiau, P.; Loth, K.; Herve, V.; et al. Structure, function, and evolution of Gga-AvBD11, the archetype of the structural avian-double-beta-defensin family. *Proc Natl Acad Sci U S A* **2020**, *117*, 337-345, doi:10.1073/pnas.1912941117.). The phylogenetic tree of birds (left) was generated with iTOL (interactive Tree Of Life, https://itol.embl.de).

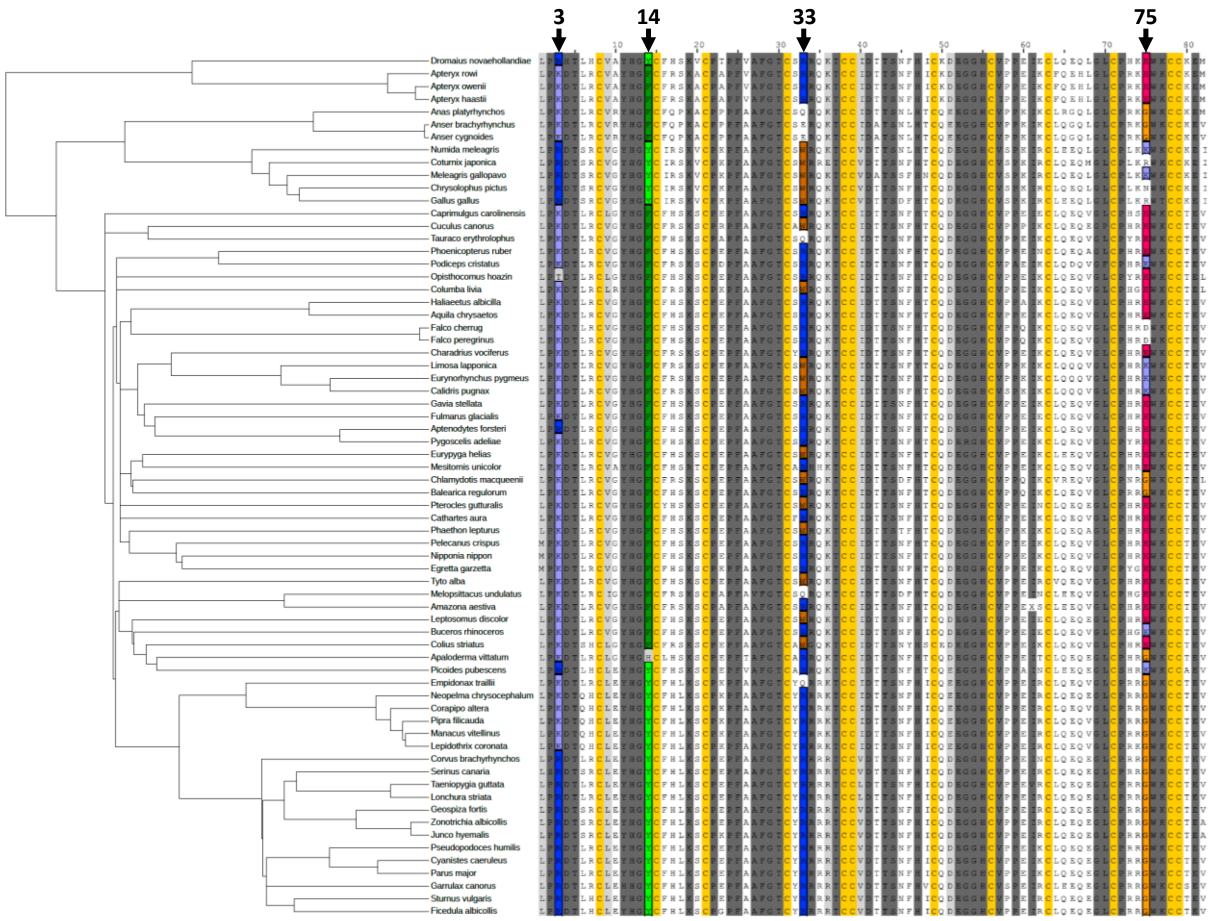

Supplement: Supplementary file 1 [file biology-11-00690-s001.zip › Figure S8.pdf]
