# Supplementary material for: The Two Domains of the Avian Double-β-Defensin AvBD11 Have Different Ancestors, Common with Potential Monodomain Crocodile and Turtle Defensins
Source: Biology (Basel). 2022 Apr 30;11(5):690. doi: 10.3390/biology11050690 (PMC9138766; doi:10.3390/biology11050690)

Figure S9. Backbone of Gga-AvBD11 (6QEU.pdb) rainbow colored from N-ter (in blue) to C-ter (in red) with disulfide bridges in sticks. Sidechains of W33 and R75 are represented in brown and blue, respectively.

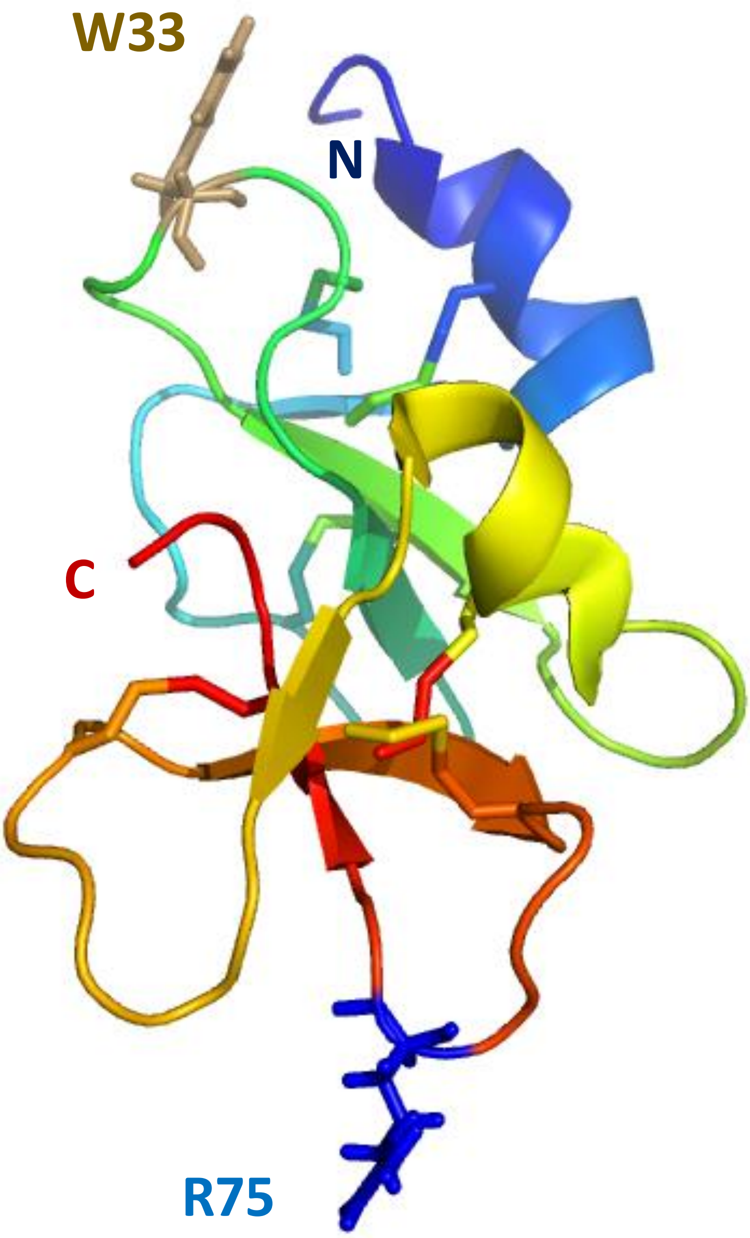

Supplement: Supplementary file 1 [file biology-11-00690-s001.zip › Figure S9.pdf]
