# Supplementary material for: The Two Domains of the Avian Double-β-Defensin AvBD11 Have Different Ancestors, Common with Potential Monodomain Crocodile and Turtle Defensins
Source: Biology (Basel). 2022 Apr 30;11(5):690. doi: 10.3390/biology11050690 (PMC9138766; doi:10.3390/biology11050690)
Supplement: Supplementary file 1 [file biology-11-00690-s001.zip › Table S1.pdf]

Table S1. List of acronyms used for animal species used in the present study.

**Acronym**	**Latin name**	**Common name**
ALLMI	*Alligator mississipiensis*	American alligator
ALLSI	*Alligator sinensis*	Chinese alligator
ANAPL	*Anas platyrhynchos*	Duck
CHESE	*Chelydra serpentina*	Common snapping turtle
CHRPI	*Chrysemys picta bellii*	Western painted turtle
CROPO	*Crocodylus porosus*	Saltwater crocodile
GALGA	*Gallus gallus*	Chicken
GAVGA	*Gavialis gangeticus*	Gharial
NIPNI	*Nipponia nippon*	Crested ibis
PELCA	*Pelusios castaneus*	West African mud turtle
TAEGU	*Taeniopygia guttata*	Zebra finch
TERCA	*Terrapene Carolina triunguis*	three-toed box turtle
