# Supplementary material for: The Two Domains of the Avian Double-β-Defensin AvBD11 Have Different Ancestors, Common with Potential Monodomain Crocodile and Turtle Defensins
Source: Biology (Basel). 2022 Apr 30;11(5):690. doi: 10.3390/biology11050690 (PMC9138766; doi:10.3390/biology11050690)
Supplement: Supplementary file 1 [file biology-11-00690-s001.zip › Table S2.pdf]

Table S2. List of protein sequences used for the construction of multiple sequence alignments (MSAs) and phylogenetic trees.

Protein name (used in this study)	Protein sequence	Source(s)
AvBD1_GALGA	GRKSDCFRKSGFCAFLKCPSLTLISGKCSRFYLCCKRIWG	Cheng et al, 2015
AvBD1_ANAPL	RNKEKCHREKGFCGFLKCSFPFIISGKCSRFFFCCKNSWRE	Cheng et al, 2015
AvBD1.1_NIPNI	ENEGQCKQEGGFCSFLKCYHPYHVFGRCSTFMVCCKR	Cheng et al, 2015
AvBD1.2_NIPNI	GNKEQCHRQKGHCALLVCQFPLIAIGKCSRFFFCCKPIWG	Cheng et al, 2015
AvBD1.1_TAEGU	TNKEQCEQENGQCSFLKCYFPYGIAGKCSRFFFCCKEMWG	Cheng et al, 2015
AvBD1.2_TAEGU	NNKKQCEREKGYCSIFNCSFPYIIAGKCSRFHFCCKKMWG	Cheng et al, 2015
AvBD1.3_TAEGU	TTKEQCQRENGYCGFLKCKYPFVIKGSCSRFFFCCKNVWG	Cheng et al, 2015
AvBD2_GALGA	RDMLFCKGGSCHFGGCPSHLIKVGSCFGFRSCCKWPWNA	Cheng et al, 2015
AvBD2_ANAPL	RDMFLCRKGSCHFGRCPIHLVRVGSCFGFRSCCKLPWDV	Cheng et al, 2015
AvBD2_NIPNI	RDMFLCRKGSCHFGRCPFHLVKVGSCFGFRSCCKDPWNA	Cheng et al, 2015
AvBD2_TAEGU	REMFLCRGGSCHFGGCPIHLIKVGRCFGFRSCCKSPWNVKEVDEPFIEE	Cheng et al, 2015
AvBD3_GALGA	ATQCRIRGGFCRVGSCRFPHIAIGKCATFISCCGRAYEVDALNSVRTSPWLLAPGNN PH	Cheng et al, 2015
AvBD3.1_ANAPL	SVLCRIRGGRCHVGSCHFPERHIGRCSGFQACCIRTWG	Cheng et al, 2015
AvBD3.2_ANAPL	HQACRRRGGFCTFGRCRFPTRPVGRCSTLVPCCRSTWG	Cheng et al, 2015
AvBD3.3_ANAPL	RQACRQRGGICTFARCRFPTTPIGRCSTAVPCCK	Cheng et al, 2015
AvBD3.4_ANAPL	DIRCTIEGGRCRFGGCQFAEKQIGKCYRIVPCCS	Cheng et al, 2015
AvBD3.5_ANAPL	TLRCRRRGGICRFGNCLFPERHIGRCSSFQPCCSRIFG	Cheng et al, 2015
AvBD3.6_ANAPL	SWLCVRRGGNCRFGRCQFAERQIGRCSAFQPCCGRTWG	Cheng et al, 2015
AvBD3_NIPNI	PFRCIQRGGYCSFGDCRYPARSIGRCSRFQQCCKNIWG	Cheng et al, 2015
AvBD3.1_TAEGU	HDKCVRRGGFCAVGGCRFPYKYVGVCSALSHCCKPIWG	Cheng et al, 2015
AvBD3.2_TAEGU	AVTCWRKQGFCAWRSCPRGARVVGICSPRMLCCK	Cheng et al, 2015
AvBD3.3_TAEGU	PLLCRQRGGFCTFGGCRFPSTPIGRCSAVQTCCKSVWG	Cheng et al, 2015
AvBD3.4_TAEGU	PLLCKRRGGYCTLGICRHGATPVGRCTPYRVCCLGGWV	Cheng et al, 2015
AvBD3.5_TAEGU	PDQCRRLRGFCFRRSCPRGSAPVGRCARGIACCKIVWG	Cheng et al, 2015
AvBD3.6_TAEGU	SARCRRRGGFCSFDGCSSPSKPIGKCSAVSVCCKSRWV	Cheng et al, 2015
AvBD3.7_TAEGU	PLLCRQRGGFCTFGGCRFPSTPIGRCSAVQTCCKSHWV	Cheng et al, 2015
AvBD3.8_TAEGU	AVTCWRKQGFCAWRSCPRGTHVVGICSPGMLCCKRLKP	Cheng et al, 2015
AvBD3.9_TAEGU	CRQRGGYCTYGRCNFPARPIGRCTLYSVCCRR	Cheng et al, 2015
AvBD4_GALGA	RYHMQCGYRGTFCTPGKCPYGNAYLGLCRPKYSCCRWL	Cheng et al, 2015
AvBD4_NIPNI	KPFMRCGYRGTFCFPGMCPRGNAYLGVCRSGHSCCKWL	Cheng et al, 2015
AvBD4_TAEGU	RPYMQCGYRGTFCHKGKCPRGNYYLGPCRSGYSCCRWL	Cheng et al, 2015
AvBD4_ANAPL	KHLMRCGYRGTFCTPGKCPRGNAYLGRCRAGHSCCKWL	Cheng et al, 2015
AvBD5_GALGA	GLPQDCERRGGFCSHRSCPPGIGRIGLCSKEDFCCRSRWYS	Cheng et al, 2015
AvBD5_NIPNI	GLPQDCERRGGFCSHGSCPPGIGRVGICSEQVFCCRRRWYP	Cheng et al, 2015
AvBD5_TAEGU	ASAQDCERRGGFCSHRSCPPGIGRIGLCSEQEFCCRMRWYP	Cheng et al, 2015
AvBD5_ANAPL	GSPQDCERRGGFCSHRSCPPGIGRIGLCSKEDFCCRRRWYP	Cheng et al, 2015
AvBD6_GALGA	SPIHACRYQRGVCIPGPCRWPYYRVGSCGSGLKSCCVRNRWA	Cheng et al, 2015
AvBD7_GALGA	RPIDTCRLRNGICFPGICRRPYYWIGTCNNGIGSCCARGWRS	Cheng et al, 2015
AvBD7_NIPNI	EPHHPCIFQKGHCFPGICRRPYYWIGTCHNGYSCCKRGWRS	Cheng et al, 2015
AvBD7_TAEGU	RLNNPCLMQNGLCFRGICRRPYYWIGTCSNGYSCCKRGWRS	Cheng et al, 2015
AvBD7_ANAPL	RPTDPCVLQNGLCFPGICRRPYYWVGTCHNGGSCCVKGWRH	Cheng et al, 2015
AvBD8_GALGA	NNEAQCEQAGGICSKDHCFHLHTRAFGHCQRGVPCCRTVYD	Cheng et al, 2015
AvBD8_NIPNI	NNDVQCKQAGGTCSTEHCNLPNTRSFGRCQQGVPCCRTVYD	Cheng et al, 2015
AvBD8_TAEGU	NTEVQCRQAGGVCSSDRCPPPHTRPFGRCQQGIPCCRTVYD	Cheng et al, 2015
AvBD8_ANAPL	NNAVQCKQAGGTCSTDHCPPPNTRAFGRCKQGIPCCRTVYD	Cheng et al, 2015
AvBD9_GALGA	ADTLACRQSHGSCSFVACRAPSVDIGTCRGGKLKCCKWAPSS	Cheng et al, 2015
AvBD9_NIPNI	ADTLACRQNRGSCSFVACTSPLVDIGTCRGGKLKCCKWTSSS	Cheng et al, 2015
AvBD9_TAEGU	ADTLACRQSRGSCSFVPCSAPLVDIGTCRGGKLRCCKWTPSS	Cheng et al, 2015
AvBD9_ANAPL	ADTVACRQSHGSCSFIACSGPLVDIGTCRNGKLKCCKWTPSS	Cheng et al, 2015
AvBD10_GALGA	DTVACRTQGNFCRAGACPPTFTISGQCHGGLLNCCAKIPAQ	Cheng et al, 2015
AvBD10_NIPNI	DTAECRSQGNFCRAGACPPTFAASGSCHGGLLKCCSKILAK	Cheng et al, 2015
AvBD10_TAEGU	DTVECRSQGRFCRAGACPPTFAATGTCHGGLLNCCSKILAE	Cheng et al, 2015
AvBD10_ANAPL	DTAACRSQGNFCRAGACPPTFAASGSCHGGLLKCCSKILAE	Cheng et al, 2015
AvBD12_GALGA	GPDSCNRDRGLCRVGNCNPGEYLAKYCFEPVILCCKPLSPTPTKT	Cheng et al, 2015
AvBD12_NIPNI	GPDSCNHEGGLCRVGNCIPGEYLARYCFEPIILCCKSLSPTAEKS	Cheng et al, 2015
AvBD12_TAEGU	GPDSCNHGGGLCRMGSCVSGEYVAQYCFEPIILCCKNPTPATTES	Cheng et al, 2015
AvBD12_ANAPL	GPDSCNHEGGLCRVGNCIPGEYLAKYCFEPVILCCKSPSTTTAKS	Cheng et al, 2015
AvBD13_GALGA	SDSQLCRNNHGHCRRLCFHMESWAGSCMNGRLRCCRFSTKQPFSNPKHSVLHTAE QDPSP	Cheng et al, 2015
AvBD13_NIPNI	SDSQQCRSNHGHCRRLCFHMERWEGSCSNGRLRCCR	Cheng et al, 2015
AvBD13_TAEGU	SDTQQCRSSRGHCRRLCFHMERWEGSCSSNGRLRCCR	Cheng et al, 2015
AvBD13_ANAPL	SDSQQCRHDHGHCRRLCFHMERWAGSCSNGRLRCCR	Cheng et al, 2015
AvBD14_GALGA	DTVTCRKMKGKCSFLLCPFFKRSSGTCYNGLAKCCRPFW	Cheng et al, 2015
AvBD14_ANAPL	DTVMCRKIKGECSFLLCSLFKRSIGTCYNGLAKCCIPF	Cheng et al, 2015
AvBD11_GALGA_Nter	LPRDTSRCVGYHGYCIRSKVCPKPFAAFGTCSWRQKTCCV	NCBI database (NP_001001779.1)
AvBD11_NIPNI_Nter	MPKDTLRCVGYHGFCFHSKSCPEPFAAFGTCSRRQKTCCI	NCBI database (XP_009465634.1)
AvBD11_TAEGU_Nter	LPRDTLRCLEYHGYCFHLKSCPEPFAAFGTCYRRRRTCCL	NCBI database (XP_002186664.2)
AvBD11_ANAPL_Nter	LPKDTLRCVRYHGFCFQPKACPPPFAAFGTCSQRQKTCCI	NCBI database (XP_005028303.2)
AvBD11_GALGA_Cter	DTTSDFHTCQDKGGHCVSPKIRCLEEQLGLCPLKRWTCCKEI	NCBI database (NP_001001779.1)
AvBD11_NIPNI_Cter	DTTSNFHTCQDEGGHCVPPEIKCLQEQVGLCPHREWKCCTEV	NCBI database (XP_009465634.1)

AvBD11_TAEGU_Cter	DTTSNFHICQDEGGHCVPPEVRCLQEQEGLCPRRGWKCCTEV	NCBI database (XP_002186664.2)
AvBD11_ANAPL_Cter	DTTSNLHTCQEEGGHCVPPKIKCLRGQLGLCPRKGWKCCKEM	NCBI database (XP_005028303.2)
OvoDA1_GALGA	LVLKYCPKIGYCSNTCSKTQIWATSHGCKMYCCLPASWKWK	Whenham et al, 2015; Zhang et al, 2019
OvoDA2_GALGA	LVLKYCPKIGYCSNTCSKTQIWATSHGCKMYCCLPASWKWK	Whenham et al, 2015; Zhang et al, 2019
OvoDA3_GALGA	LVLKYCPKIGYCSNTCSKTQIWATSHGCKMYCCLPANWKWK	Whenham et al, 2015; Zhang et al, 2019
OvoDA1_NIPNI	QIRKHCPKVGYCSSKCTKVDVWSFSADCKYYCCIPPGWQGK	Zhang et al, 2019
OvoDA2_NIPNI	QIRKQCPKVGYCSSKCNKADLWSFSADCKYYCCIPPGWKGK	Zhang et al, 2019
OvoDA1_TAEGU	KFRKTCAPMGYCSPKCRVMDLKYTSGDCKYSCCIPTAWKGK	Whenham et al, 2015; Zhang et al, 2019
OvoDA2_TAEGU	KFRKTCAPMGYCSPKCRVMDLKYTSGDCKYSCCIPTAWKGK	Whenham et al, 2015; Zhang et al, 2019
OvoDBalpha_GALGA	APGYRKRKGTCKGYCAPTCNKKDEWSFHQSCKKMYCCLLPLKKGK	Zhang et al, 2019
OvoDBalpha_NIPNI	QLKGACGGYCSYTCAKRDEWTFSQSCGKMYCCIPPPKKGK	Zhang et al, 2019
OvoDBalpha_TAEGU	AQGFCDGYCAHACDETEEWSFNPYCEELHCCIPSPKKGK	Zhang et al, 2019
OvoDBbeta_GALGA	QSKKCCGRCSSRMCTKREKEEHTEDCRGSFCCLTHRKKK	Zhang et al, 2019
OvoDBbeta_NIPNI	QSKKSCSGYCSRTCAKGEKEEHTEDCRRMYCCLTHRKKK	Zhang et al, 2019
OvoDBbeta_TAEGU	QPKRSCRGHCSRTCGKGEREEHTEDCGGMHCCLTHRKRK	Whenham et al, 2015; Zhang et al, 2019
OvoDA1_ANAPL	QVRKYCPKVGYCSSKCSKADVWSLSSDCKFYCCLPPGWKGK	Whenham et al, 2015
OvoDB1_ANAPL	QKKGFCAGYCSYSCAKTDEWTFHQTCGKMYCCIPPPKKGK	Whenham et al, 2015
BD1_GAVGA	QHKAQEEAQDPALQDEAEAVMAAPENTPISRSSCRRSGATCRVGFCFGGELKLGSC AFLRPCCKELPGL	Santana et al, 2021
BD2_GAVGA	QDMAVAQDEAEPQDLDEMGEEAETKVMEAEDAAGMDFPGLNLGESPAHCRWNR GICRRTYCKKHDRNCRYNPCRIQERRAGWCLSSHVCCVKALL	Santana et al, 2021
BD3_GAVGA	QDIQAQNKAEIQELNNPAQLRRRKFCFRRGVCKSRCSRNEDSARRCRNRQHCCVK RRHH	Santana et al, 2021
BD4_GAVGA	QAQAQDVVATQGEAEAQDLDEVDEEAEDNAMEAEYAARMGSPDVKPQEFPLVC RVLLGVCRFSRCQKNERTIGSCSSRRACCKRH	Santana et al, 2021
BD5_GAVGA	FFQIYGNTKLCKLSGGSCFLRSCPRKFVSFGTCTRECMCCIRVRRVRD	Santana et al, 2021
BD8_GAVGA	FPKIGYFHCQSRKGQCFQHTCPPNTKYIGSCNQLGNCCQR	Santana et al, 2021
BD9_GAVGA	LVTVMAQGEAEPQDLGEMQEQAEDNIMEAEDSDYKGSADLKPLASPLWCGWKG GYCQHHCKKEEHKTGWCTMNYVCCH	Santana et al, 2021
BD10_GAVGA	FVDVAPADTIACRNQGNFCRLRTCPPTFEGTGTCHNGALLCCSKVPGS	Santana et al, 2021
BD12_GAVGA	SKHICRTAGGQCRMGTCLSGEVRIGDCFSPVILCCKKYPVRKTTRELQRGA	Santana et al, 2021
BD13_GAVGA	TYYSTLQCRNNHGHCRRLCFHGEQWIGNCNGRHQHCCK	Santana et al, 2021
BD14_GAVGA	DTLTCMKNNGTCSFMLCPIFMKAIGTCYDGEAKCCRRCI	Santana et al, 2021
BD15_GAVGA	QPYRSCLDMGGRCVRYDICHPNFVINARCPHRTVCCRRY	Santana et al, 2021
BD17_GAVGA	LGRCNLLNGVCRHTLCHSLEKYVGRCHRGLRNCCVDDYVLKYKM	Santana et al, 2021
BD18_GAVGA	LPFPITKLSLSSHPADLKPHGSPTDCHRQLGICRCFICNLFEITIGSCNRNHVCCRRWI	Santana et al, 2021
BD19_GAVGA	QQYRDCKDRGGDCILHDTCLSTGEVIYAPCPRWLICCRRLW	Santana et al, 2021
BD20_GAVGA	QRATRYVNHCLQKGGTCRYDDCEAGEEQIGTCYRQTMVCCRDEE	Santana et al, 2021
BD21_GAVGA	LPILSLLQCLNLQGTCLFSVSFCSGITIRLLGCNCCSP	Santana et al, 2021
BD23_GAVGA	IPSCRFSGGHCIWNWERCRSGRFLAAPCPFRKRCCKS	Santana et al, 2021
BD1_CROPO	QHKAQEEAQDPALQDEAEAVMAAPENTPISRSSCRRSGATCRVGFCFGGELRLGSC AFLRPCCKELPGL	Santana et al, 2021
BD2_CROPO	QDVVVAQDKAEPQDLDEMEEEAETEVMEAQDAAGMDFPGLNLGESPAHCRWRR GICRPTHCKKNDPNCRYNPCRFQERIVGWCLSSHVCCVKAKL	Santana et al, 2021
BD3_CROPO	QDIQAQNKAEIQELNNPAQPRRRKFCFRRGVCKSRCSRNEDSARRCRNRQHCCIKR RH	Santana et al, 2021
BD4_CROPO	QDVVVTQGEAEAQDLDEMDEEAKDNAMEAEYAARMGSPDVKPQEFPVVCRILLG VCRFSRCRKNERTIGSCSSSRACCKRR	Santana et al, 2021
BD5_CROPO	FFQIYGNTKLCKLNGGSCFLRSCPRKFVSFGTCTRECMCCIR	Santana et al, 2021
BD8_CROPO	FPKIGYFHCRSQNGNCYQYACPPNTKYIGSCNKLGNCCQRV	Santana et al, 2021
BD9_CROPO	QVTVVAQGEAEPQDLGEKQEQAEDNIMEAEDAGYKGSADLKPLPSPLWCGWKGG YCRHHCKKEERKTGWCTTNYVCCH	Santana et al, 2021
BD10_CROPO	FVDVAPADTVACRNQGNFCRLGTCPPTFEGTGTCNNGALLCCSKVPGL	Santana et al, 2021
BD12_CROPO	SKHVCRTAGGQCRMGICLSGEVRIGDCFIPVILCCKKYPVRKETGELQGGA	Santana et al, 2021
BD13_CROPO	SYYSTLQCRNNHGHCRRLCFHGEQWIGNCNGRHQHCCK	Santana et al, 2021
BD14_CROPO	DTLTCTKNNGTCAFMLCPIFMKAIGTCYDGAAKCCRRCI	Santana et al, 2021
BD15_CROPO	SRSCLDRGGRCIRYNTCHPNLIINARCPHQTVCCRRR	Santana et al, 2021
BD17_CROPO	LGRCNLLNGVCRHTLCHSLEKYVGRCHRGLRNCCVDDYVLKYKM	Santana et al, 2021
BD18_CROPO	QDLKPHGSPTDCHRKLGICRHVFCNLFEITIGYCNRHHVCCRRWI	Santana et al, 2021
BD19_CROPO	QQYHDCKDRGGDCILHDTCLSSGEVIYAPCPRWLICCRRLR	Santana et al, 2021
BD20_CROPO	QRATRYVNHCLQKGGTCRYDDCEAGEEQIGTCYRQTMVCCRDEE	Santana et al, 2021
BD21_CROPO	LPILSFLQCLNLQGTCLLTVGFCNGITIRLLGCDCCTP	Santana et al, 2021
BD23_CROPO	SPSCRSFGDHCIWNWERCRSGRFLAVPCPFRKRCCKS	Santana et al, 2021
BD1_ALLMI	QHKAQEEAQDPALQDEAEAVMAAPENTPISRSNCKRSGATCRVGFCFGGEIKLGSC AFLRPCCKELPGL	Santana et al, 2021
BD2_ALLMI	QDVVVAQDEAEPQDLGEMEEEAETEVMEAEDATGMDFPGPKLGESPAHCRWKRG VCRRTHCKRNDRNCRHTPCKPAERIIGWCLSTYVCCRKAYL	Santana et al, 2021

BD3_ALLMI	QDIQAQDKAEIQELNHPAQPRRRKFCSRQGVCKPRCSGNENSSRRCRNHQRCCVKRRQ	Santana et al, 2021
BD4_ALLMI	QDVVVAQDEAEAQDLDDIDEEAQDNAMEAEYAATMGSPDVKPQEYPVVCRVLLGVCRPFRCLRNERTIGSCSSNHACCKRY	Santana et al, 2021
BD5_ALLMI	FFQIYWNTKLCKLNGGSCFLRSCPRQFVSFGTCTQECMCCIRHRRTRD	Santana et al, 2021
BD6_ALLMI	MPNPVGEKDPQKEADTWDEVEDDVGEEEGDVEAQGRGENSPMICGFSGGSCRTGCSSNEVMAGKCYGSYLCCIPR	Santana et al, 2021
BD7_ALLMI	MPNPVGEKQPHKEADTWDGVEDDASKAKGNVEAEGAGGENNPMVCSYSGGSCRQRCIGHEVMVGKCYGTFICCVHM	Santana et al, 2021
BD8_ALLMI	FPQIGYFHCQQNKGQCFQHICPPNTKYIGSCKQLGNCCQRV	Santana et al, 2021
BD9_ALLMI	QVTVVAGEAEPQDLGEMQEQAEDNVMDAEDADDKGSADLPLASPLWCGWKGGYCRHHCKEKERKTGLCTVNYVCCL	Santana et al, 2021
BD11_ALLMI	QDGAVAQDEAEAQDLDEMEEEAEDEFVEAEDAAGMGSPELARKDRPRCRKGLFCRPKCGQKEHVIGTCPKGLICCRIL	Santana et al, 2021
BD12_ALLMI	SKNVCRSAGGQCQMGTCLSGEVRIGDCFTPVILCCKKYLARKTPGELQGGA	Santana et al, 2021
BD13_ALLMI	SYYSTLQCRNNHGHCRRLCFHRERWIGNCNGGHQHCCK	Santana et al, 2021
BD14_ALLMI	DTLTCTKNNGTCSFMLCPIFMKAIGSCYDGAAKCCRRCI	Santana et al, 2021
BD15_ALLMI	YRECRNRGGECRPHGSCHPGSVIPVRCPHRTVCCRRR	Santana et al, 2021
BD16_ALLMI	MPNPVGENDPQVEADTWDEVEDDAGEAEGDVEAEGAGGENSPMICGFSGGSCRTVCLISEVMAGKCYSSYLCCLPR	Santana et al, 2021
BD17_ALLMI	LGRCNLLNGVCRHTLCHSLEKYIGRCHRGLRNCCVDDYVLKYKM	Santana et al, 2021
BD18_ALLMI	LKPQGSPTDCHRQLGVCRSFLCFFFETTIGSCNRHQVCCRRWI	Santana et al, 2021
BD19_ALLMI	QEYHDCKNRGGDCILHDTCLSTGEIIYAPCPRWLICCKRLR	Santana et al, 2021
BD20_ALLMI	QRASRYVNHCLQKGGTCRYDDCEAGEEQIGTCYRQTMVCCRDEE	Santana et al, 2021
BD21_ALLMI	LPILSLIQCLNLGGICLISVSLCDGVTIRLLGCNCCSSR	Santana et al, 2021
BD23_ALLMI	EEVIPSCRFSGGYCIWNWERCRSGHFLVALCPFRKRCCKS	Santana et al, 2021
BD1_ALLSI	QHKAQEEAQDPALQDEAEAMLAAPENSPISRSNCKRSGAICRVGFCFGGEVKLGSCAFLRPCCKELPGL	Santana et al, 2021
BD2_ALLSI	QDVVVAQDEAEPQDLDEMEEEAETEVMEAEDATGMDFPGPKLGESPAHCRWKRGVCRPTHCKRNDSNCRHTPCKPEERIIGWCLSTHVCCRKALL	Santana et al, 2021
BD3_ALLSI	QDIPAQDKAEIQELNHPAQPRRRKFCSRRGVCKSRCSGNENSSRRCRNRQRCCVKRRQ	Santana et al, 2021
BD4_ALLSI	QDVVVAQGEAEAQDIDDMDEEVQDNAMEAEYAASMGSPDVKPQEYPIVCRVLLGVCRPFRCLRNEHTIGSCSSNHACCKRH	Santana et al, 2021
BD5_ALLSI	FFQIYGNTKLCKLNGGSCFLRSCPRQFVSFGTCTRECMCCIRRTRD	Santana et al, 2021
BD6_ALLSI	MPNPVGEKDPEKEADIWDEVKDDAGEEEGDVEAEGAEGENSPMICGFSGGSCRTSCSSNEVMAGKCYGSYLCCVPR	Santana et al, 2021
BD7_ALLSI	NPVGEKQPQKEADTWDGVEDDASETKGDVEAEGAGGQNNPMICSYSGGSCRPRCTGHEVMVGKCYGTFICCVRM	Santana et al, 2021
BD8_ALLSI	FPQIGYFHCQQNKGQCFKHICPPNTKYIGSCKQLGNCCQRV	Santana et al, 2021
BD9_ALLSI	QVTVVAQGEAEPQDLGEMQEQAEDNVMDAEDADDKGSADLKPLASPLWCGWKGGYCRHHCKEKERKTGLCTVNYVCCL	Santana et al, 2021
BD10_ALLSI	FVDVAPADTVACRSQQNFCRLGTCPPTFEASGTCHNGALLCCSKVPGV	Santana et al, 2021
BD11_ALLSI	QDGAVAQDESEAQDLDEMEEEAEDEFVEAEDVAGMGSPELARKDRPRCRKGLFCRPKCGRKEHVIGTCPKGLICCRIL	Santana et al, 2021
BD12_ALLSI	SKNVCRSAGGQCQMGTCLSGEVRIGDCFTPVILCCKKYLARKTTGELQGGA	Santana et al, 2021
BD13_ALLSI	SYYSTLQCRSNHGHCRRLCFHGERWIGNCNGGHQHCCK	Santana et al, 2021
BD14_ALLSI	DTLTCTKNNGTCSFMLCPIFMKAIGTCYDGAAKCCRRCI	Santana et al, 2021
BD15_ALLSI	SRECWNRGGDCRPHGSCRPGSVIPVRCPYRTVCCRRR	Santana et al, 2021
BD16_ALLSI	MPNPVGENDPQKEADTWDEVEDDAWEAEGDVEAAGASGENSPMICGFSGGSCRTVCLISEVMAGKCYGSYVCCVPR	Santana et al, 2021
BD17_ALLSI	LGPCNLLNGVCRHTLCHSLEKYVGRCHHGLRNCCVDDYVLKYKM	Santana et al, 2021
BD18_ALLSI	LKPHGSPTDCHTQLGVCRRFLCFPFEKTIGSCNRQQVCCRRWI	Santana et al, 2021
BD19_ALLSI	QEYHDCKNRGGDCILHDTCLSTGEIIYAPCPRWLICCRRLR	Santana et al, 2021
BD20_ALLSI	QRASRYVNHCLQKGGTCRYDDCEAGEEQIGTCYRQTMVCCRDEE	Santana et al, 2021
BD21_ALLSI	LPILPLIQCLNLGGICLISVSLCDGITIRLLGCNCCSSH	Santana et al, 2021
BD22_ALLSI	QHKVQEEAQDPALQDEAEAVMAAPESTPISRSHCRCSGTMCQTEFCFRGEVKLGSCTFLHPCCKELLG	Santana et al, 2021
ENSCPBG00000020207_CHRPI	ECLSNHGHCRRLCFHMEHQVGTCTNGHLRCCK	Ensembl database
ENSCPBG00000020212_CHRPI	QGSEASCRRTGGLCLVSKCTQAEHHVEYCLTPVILCCKRLPVST	Ensembl database
ENSCPBG00000020214_CHRPI	TLLHLIDSLACKGKHGRCRKAFCFLNERRIGMCTFSRRFCCRRKK	Ensembl database
ENSCPBG00000020216_CHRPI	LPADTLRCISNNGLCHQTLCPGTLFEFGTCSHGRATCCKGRW	Ensembl database
ENSCPBG00000020217_CHRPI	EDQDEPQEPLTGNSHGLYQSKLTVPVSLSFVNGNLSFLFAEIRSSCFASGGQCRHGLCPWKEMKIASCGFARPCCKKVI	Ensembl database
ENSCPBG00000020219_CHRPI	DFLDNINCRSNFGFCHSGDCPISTTLIGTCINGKINCCKRTTTP	Ensembl database
ENSCPBG00000020224_CHRPI	DVGPPPADTLACKAQGGFCRLLNCPPVFSVSGTCHGGQLQCCTSVVPSQSME	Ensembl database
ENSCPBG00000020230_CHRPI	QNIIQCIRLGGSCRSGSCPSGFARIGTCSGSDSCCLLREPWYHP	Ensembl database
ENSCPBG00000020233_CHRPI	FTCSPGATMRCLQNGGRCYPWQCPPNTYNIGRCCPWRLCCRRVS	Ensembl database
ENSCPBG00000020235_CHRPI	QGINTPFACRRAGGFCRRGRCPPNFRRIGSCGFGQSCCKRGWVSSGCHKGDITV	Ensembl database
ENSCPBG00000020239_CHRPI	EFINSSRACRRARGSCFRVCFRRYRLIGTCGQGLSCCRTWVSSGSTKLISQC	Ensembl database
ENSCPBG00000020243_CHRPI	MSHDNGIHLKLVAFSFRRWGFTQFIDKRRACIFSGGFCRGRCPPNFRRIGSCSFGQSCSFSIKKTAILIPL	Ensembl database
ENSCPBG00000020248_CHRPI	CIEYGGLCFGACPPPFRGIGSCGGGVSCCVWRVSSGFHKANVTV	Ensembl database
ENSCPBG00000003006_CHRPI	EHILSDTVMCRNTQGKCSFAICPFFTNANGTCYNGKAKCCRPYL	Ensembl database
ENSCPBG00000003028_CHRPI	QNFNINCIRKGGFCFFWRCPQNWKLIGFCSNGYVCCIRRRWGLGSEDLGS	Ensembl database
ENSCPBG00000003054_CHRPI	YDLSRNCRLRGGICYIGICPRRMFRSGSCSRGNVCCLSCVWYNYTGV	Ensembl database
ENSCPBG00000003079_CHRPI	TEVSDRGIIGTAVCLSKKGACFLFHCPLNTMRIGRCGLFWHCCRW	Ensembl database
ENSTMTG00000011266_TERCA	LPADTLRCINNNGFCYQTRCPGTLFEFGTCSHGRATCCKGRW	Ensembl database
ENSCSRG00000018998_CHESE	LNNGGLTLPLAGLISHHFSDTPVPPAGLSLPADTLRCVSNNGLCHQTLCPRPLFALGTCSYGRTTCCKGRW	Ensembl database
ENSPCEG00000007416_PELCA	YLLKLIDSFTCKRKHGMCREDFCYLNEKQIGTCPLSRWYCCRRKK	Ensembl database

References:

Cheng, Y.; Prickett, M.D.; Gutowska, W.; Kuo, R.; Belov, K.; Burt, D.W. Evolution of the avian beta-defensin and cathelicidin genes. *BMC Evol Biol* **2015**, *15*, 188, doi:10.1186/s12862-015-0465-3.

Santana, F.L.; Estrada, K.; Ortiz, E.; Corzo, G. Reptilian beta-defensins: Expanding the repertoire of known crocodylian peptides. *Peptides* **2021**, *136*, 170473, doi:10.1016/j.peptides.2020.170473.

Whenham, N.; Lu, T.C.; Maidin, M.B.; Wilson, P.W.; Bain, M.M.; Stevenson, M.L.; Stevens, M.P.; Bedford, M.R.; Dunn, I.C. Ovodefensins, an Oviduct-Specific Antimicrobial Gene Family, Have Evolved in Birds and Reptiles to Protect the Egg by Both Sequence and Intra-Six-Cysteine Sequence Motif Spacing. *Biol Reprod* **2015**, *92*, 154, doi:10.1095/biolreprod.114.126839.

Zhang, L.; Chen, D.; Yu, L.; Wei, Y.; Li, J.; Zhou, C. Genome-wide analysis of the ovodefensin gene family: Monophyletic origin, independent gene duplication and presence of different selection patterns. *Infect Genet Evol* **2019**, *68*, 265-272, doi:10.1016/j.meegid.2019.01.001.
