# Supplementary material for: The Two Domains of the Avian Double-β-Defensin AvBD11 Have Different Ancestors, Common with Potential Monodomain Crocodile and Turtle Defensins
Source: Biology (Basel). 2022 Apr 30;11(5):690. doi: 10.3390/biology11050690 (PMC9138766; doi:10.3390/biology11050690)
Supplement: Supplementary file 1 [file biology-11-00690-s001.zip › Table S3.pdf]

Table S3. List of nucleotide sequences used for the positive selection analysis.

Bird species	Accession (NCBI or Ensembl)	Nucleotide sequence (mature region)	Protein accession (NCBI or Ensembl)
Apteryx rowi	XM_026061083.1	TTGCCTAAAGACACCTTACGTTGTGTTGCCTACCATGGTTTCTGCTTCCGTTC<br>AAAAGCCTGCCCAGCACCTTTTGTTGCATTTGGAACTTGCTCTCGGCGTCAGA<br>AAACCTGCTGCATAGACACTACATCAAACTTCCATATTTGTAAAGATGAGGG<br>GGGTCATTGCGTACCTCCAGAAATCAAATGTTTCCAAGAACATCTGGGACTT<br>TGCCCTCGCAAAGAATGGAAGTGCTGCAAAGAAATGCAA	XP_025916868.1
Dromaius novaehollandiae	XM_026113496.1	TTGCCTAGACACACCTTACATTGTGTTGCCTACCATGGTTACTGCTTCCATTC<br>AAAAGTCTGCCCAACGCCTTTTGTTGCATTTGGAACTTGCTCTCGGCGTCAGA<br>AAACCTGCTGCATAGATACTACATCAAACTTCCATATTTGTAAAGATGAGGG<br>GGGTCATTGCGTACCYCCAGAAATCGAATGTCTTCAAGAACAGCTGGGACTT<br>TGCCCTCACAAAGAATGGAAGTGCTGCAAGGAAATGCAA	XP_025969281.1
Chauna torquata	VXAL01001524.1	TTGCCCAAAGACACCTTACGTTGTGTTGGCTACCATGGTTTCTGCTTCCGCTC<br>AAAAGTCTGCCCAACACCATTTGCTGCATTTGGAACTTGCTCTTGGCGTCAGA<br>AAACCTGCTGCATAGACACTACATCAAACTTGCATACCTGTCAAGATGAAGG<br>GGGCCATTGTGTACCTCCGAAAATCAGATGTCTGCAAGAACAGCTGGGACTT<br>TGCCCTCACAGAGAATGGAAGTGCTGCAAAGAAGTG	NXK44927.1
Anseranas semipalmata	VXAA01000532.1	TTGCCCAAAGACACCTTACGCTGTGTTCGCTACCATGGTTTCTGCTTCCATTC<br>AGAAGTCTGCCCAACACCATTTGCTGCATTTGGAACTTGCTCTCGTCGTCAGA<br>AAACCTGCTGCATAGACACTACATCAAACTTCCATACTTGTCAAGATGAAGG<br>GGGCCATTGCGTACCTCCAAAAATCAAATGTCTGCAAGAACAGCTGGGACTT<br>TGCCCTCACAAGGAATGGAAGTGCTGCAAAGAAATG	NXI61744.1
Anser cygnoides domesticus	XM_013184427.1	TTGCCCAAAGACACCTTACGTTGTGTTCGCTACCACGGTTTCTGCTTCCAACC<br>AAAAGCCTGCCCACCGCCATTTGCTGCATTCGGAACTTGCTCTGAACGTCAG<br>AAAACCTGCTGCATAGACGCTACATCAAACCTCCATACTTGTCAAGAAGAAG<br>GGGGCCATTGCGTACCTCCGAAAATCAAATGTCTGCAAGGGCAGCTGGGACT<br>TTGCCCTCGCAAAGGATGGAAGTGCTGCAAAGAAGTGCAA	XP_013039881.1
Cygnus atratus	XM_035563646.1	TTGCCCAAAGATACCTTACGTTGTGTTCGCTACCACGGTTTCTGCTTCCAACC<br>AAAAGCCTGCCCACCGCCATTTGCTGCATTCGGAACTTGCTCTGAACGTCAG<br>AAAACCTGCTGCATAGACGCTACATCAAACCTCCATACTTGTCAAGAAGAAG<br>GGGGCCACTGCGTACCTCCGAAAATCAAATGTCTGCAAGGGCAGCTGGGACT<br>TTGCCCTCGCAAAGGATGGAAGTGCTGCAAAGAAGTGCAA	XP_035419539.1
Anas platyrhynchos	XM_005028246.4	TTGCCCAAGGACACCTTACGTTGTGTTCGCTACCACGGTTTCTGCTTCCAACC<br>AAAAGCCTGCCCACCGCCGTTTGCTGCATTCGGAACTTGCTCTCAACGTCAG<br>AAAACCTGCTGCATAGACACTACATCAAACCTCCATACTTGTCAAGAAGAAG<br>GGGGCCATTGCGTGCCTCCGAAAATCAAATGTCTGCGAGGGCAGCTGGGACT<br>TTGCCCTCGCAAAGGATGGAAGTGCTGCAAAGAGATG	XP_005028303.2
Odontophorus gujanensis	VXAB01005760.1	TTGCCCAGAGACACCTCACGTTGTGTTGGCTACCACGGGTACTGCATCCGTTC<br>CAAAGTCTGTCCCAAACCATTTGCTGCTTTTGGAACTTGCTCTTGGCGTCAGA<br>AAACCTGCTGCGTAGATACTACATCAGACTTCCATACTTGTCAAGATGAGGG<br>GGGCCATTGCGTATCTCCAAAAATCAGATGCCTGCAAGAACAGCGGGGACTT<br>TGCCCTCTAAAAAAATGGAAGTGCTGCAGAGAAATA	NXJ08707.1
Meleagris gallopavo	ENSMGAT00000015987.1	TTGCCCAGAGACACCTCACGTTGTGTTGGCTACCATGGGTACTGCATCCGTTC<br>CAAAGTCTGCCCCAAACCGTTTGCTGCATTTGGAACTTGCTCTTGGCGTCAGA<br>AAACCTGCTGTGTAGACGCTACATCAAGCTTCCATAATTGTCAAGACGAGGG<br>AGGCCATTGTGTATCTCCAAAAATCAGATGTCTGCAAGAACAGCTGGGACTT<br>TGCCCTCTGAAAAAATGGAAGTGCTGCAAAGAAATA	ENSMGAP00000015039.1
Coturnix japonica	XM_015859649.2	TTGCCCAGAGACACCTCACGTTGTGTTGGCTACCACGGGTACTGCATCCGTTC<br>CAAAGTCTGCCCAAAACCATTTGCTGCATTTGGAACTTGCTCTTGGCGTCGGG<br>AAACCTGCTGTGTAGACACCACATCAAACTTCCATACCTGTCAAGACGAGGG<br>GGGCCATTGTGTATCTCCAAAGATCAGATGTTTGCAAGAACAGATGGGACTC<br>TGCCCTCTAAAAAGATGGAAGTGCTGCAAAGAAATA	XP_015715135.1
Gallus gallus	NM_001001779.1	TTGCCCAGAGACACCTCACGTTGTGTTGGCTACCATGGGTACTGCATCCGTTC<br>CAAAGTCTGCCCCAAACCATTTGCTGCATTTGGAACATGCTCTTGGCGTCAGA<br>AAACCTGCTGCGTAGACACTACATCAGACTTCCATACTTGTCAAGACAAGGG<br>GGGCCATTGCGTATCTCCAAAAATCAGATGTCTGGAAGAACAGCTGGGACTT<br>TGCCCTCTAAAAAGATGGACGTGCTGCAAAGAAATA	NP_001001779.1
Columba livia	XM_005513638.2	TTGCCCAAAGACACCTTACGTTGTCTCAGATACCACGGCTTCTGCTTCCGATC<br>CAAATCCTGCCCAAAGCCATTTGCTGCATTTGGAACCTGCTCTTGGCGTCAGA<br>AAACCTGCTGCATAGACACGACATCAAACTTCCATACTTGTCAAGATGAGGG<br>AGGTCATTGTGTGCCTCCAGAAATCAAATGTCTGCAAGAACAAGTGGGACTT<br>TGCCCTCACGGAGAATGGAAGTGCTGCACAGAATTG	XP_005513695.1
Aramus guarauna	VXBL01010431.1	TTGCCCAAAGACACCTTACGTTGTGTTGGATATCATGGTTTCTGCTTCCATTC<br>AAAATCCTGCCCAGAGCCGTTTGCTGCGTTTGGAACTTGCTCTCGGCGCCAG<br>AAAACCTGCTGCATAGACACGACATCAAACTTCCATACCTGTCAAGACGACG<br>GGGGTCATTGCGTACCTCCAGAAATCAAATGTCTGCAAAAGCAGATGGGACT<br>TTGCCCTCACAGAGGATGGAAGTGCTGCACAGAAGTG	NXO60882.1
Charadrius vociferus	KL872452.1	TTGCCCAAAGACACCTTACGTTGTGTTGGATACCATGGTTTCTGCTTCCGTTC<br>AAAATCCTGCCCAGAGCCATTTGCTGCGTTTGGAACTTGCTATCGGCGTCAG<br>AAAACCTGCTGCATAGACACAACATCGAACTTCCATACTTGTCAAGACGAGG<br>GGGGTCATTGCGTACCTCCAGAAATCAAATGTCTGCAAGAACAAGTGGGACT<br>TTGCCCTCACAGAGAATGGAAGTGCTGCACAGAAGTG	KGL95683.1
Rostratula benghalensis	VXAI01000254.1	TTGCCCGAAGACACCTTACGTTGTGTTGGATACCATGGTTTCTGCTTCCATTC<br>AAAATCCTGCCCAGGGCCATTTGTTACATTTGGAACTTGCTCTCGGCGTCGGA<br>AAATGTGCTGCATAGACACAACATCAAACTTCCATACGTGTCAAGCTGAGGG<br>GGGTCACTGTGTACCTCCAGAAATCAAATGTCTGCAGCAACAAGTGGGACTC<br>TGCCCTCACAGGAAATGGAAGTGCTGCACAGAACTG	NXJ68321.1
Pedionomus torquatus	VZRU01010140.1	TTGCCCAAAGACACCTTACGTTGTGTTGGATACCACGGTTTCTGCTTCCGTTC<br>AAAATCCTGCCCAAAGCCATTTGCCGCATTTGGAACTTGCTCTTGGCGTCAGA<br>AAACCTGCTGCATAGACACGACATCAAACTTCCATACTTGTCAAGATGAGGG<br>GGGTCACTGTGTACCTCCAAAAATCAAATGTCTGCAACAGCAAGTGGGACTT<br>TGCCCTCACAGGAAATGGAAGTGCTGCACAGAAGTG	NWW48347.1

Thinocorus orbignyianus	VXBW01005935.1	TTGCCCAAAGACACCTTACGTTGTGTTGGATACCGTGGTTTCTGCTTCCGTTC<br>AAAATCCTGCCCACGGCCGTTTGCTGCATTTGGAACTTGCTCTTGGCGTCAGA<br>AAACCTGCTGCATAGACACGACATCAAACTTCCATACTTGCCAAGATGAGGG<br>GGGTCACTGTGTGCCTCCAAAAATCAAATGTCTGCAACAGCAAGTGGGACTT<br>TGCCCTCACAGGAAATGGAAGTGCTGCACAGAACTG	NXP11758.1
Arenaria interpres	VXAK01013874.1	TTGCCCAAAGACACCTTACGTTGTGTTAGATACCATGGTTTCTGCTTCCGTTC<br>AAAATCCTGCCCAGAGCCATTTGCTGCGTTTGGAACTTGCTCTTGGCGTCAGA<br>AAACCTGCTGCATAGACACGACATCAAACTTCCATACTTGTCAAGACGAAGG<br>GGGTCACTGTGTACCTCCAAAAATCAAATGTCTGCAACAACAAGTGGGACTT<br>TGCCCTCACAGGAAATGGAAGTGCTGCACAGAAGTG	NXK22639.1
Calidris pugnax	XM_014948388.1	TTGCCCAAAGACACCTTACGTTGTGTTGGATACCACGGTTTCTGCTTCCGTTC<br>AAAATCCTGCCCAGAGCCATTTGCTGCGTTTGGAACTTGTTCTTGGCGTCAGA<br>AAACCTGCTGCATAGACACGACCTCAAACTTCCATACTTGTCAAGACGAAGG<br>GGGTCACTGTGTATCTCCAAAAATCAAATGTCTGCAACAACAAGTGGGACTT<br>TGCCCTCACAGGAAATGGAAGTGCTGCACAGAAGTG	XP_014803874.1
Sula dactylatra	WEKW01013102.1	ATGCCCAAAGACACCTTACGCTGTGTTGGATACCACGGTTTCTGCTTCCATTC<br>AAAATCCTGCCCAGAGCCATTTGCCGCGATTGGAACTTGCTCTCGGCGTCAG<br>AAAACCTGCTGCATAGACACGACATCAAACTTCCATACTTGTCAAGACGAGG<br>GGGGTCATTGTGTACCTCCAGAAATCAAATGTCTGCAAGAACAAGTGGGACT<br>TTGCCCTCACAGAGAATGGAAGTGCTGCACAGAAGTG	NWI27162.1
Pelecanus crispus	XM_009490075.1	ATGCCCAAAGACACCTTACGTTGCGTTGGATACCATGGTTTCTGCTTCCATTC<br>AAAATCCTGCCCAGAGCCGTTCGCCGCGTTTGGAACTTGCTCTCGGCGTCAG<br>AAAACCTGCTGCATAGACACGACATCAAACTTCCATACTTGTCAAGATGAGG<br>GGGGTCATTGTGTACCTACAGAAATCAAATGTCTGCAAGAACAAGTGGGACT<br>TTGCCCTCATAGAGAATGGAAGTGCTGCACAGAAGTG	XP_009488350.1
Colius striatus	XM_010204885.1	TTGCCCAAAGACACCTCGCATTGTCTTGGATATGAAGGTTTCTGCTTCCGATC<br>AAAATCCTGCCCAGAGCCATTTGCTGCATTTGGAACTTGTGCTTGGGGTCAG<br>AAAACCTGCTGCATAGACACAACATCAAACTACCATAGTTGTGAAGACAAGG<br>AGGGTCATTGCGTGCCTCCAGAAATCAAATGTCTGCAAGAACAAGTGGGGCT<br>CTGCCCTCACAGAGAATGGAAGTGCTGCACAGAAGTG	XP_010203187.1
Trogon melanurus	VXAG01001209.1	TTGCCCAAAGACACCTTACGTTGTCTTGGATACCACGGTTATTGCTTCCATTC<br>AAAATCCTGCCCAGAACCATTCGCCGCGTTTGGAACCTGCTCTCGGCGTCAG<br>AAAACCTGCTGCATAGACACGACATCAAATTTCCATACTTGTCAAGATGAGG<br>GGGGTCATTGTGTACCTCCAGAAATCAAATGTCTGCAAGAACAAGTGGGACT<br>TTGCCCTCACAGAGGATGGAGGTGCTGCACAGAAGTG	NXJ83125.1
Buceros rhinoceros silvestris	KL517598.1	TTACCCAAAGACACCTTACGTTGTGTTGGATACCATGGTTTCTGCTTCCATTC<br>AAAATCCTGCCCAGAGCCGTTTGCCGCGTTTGGAACTTGTTCTCGGCGTCAGA<br>AAACCTGCTGCATCGACACGACATCAAACTTCCATATCTGTCAAGATGAGGG<br>GGGTCATTGTGTACCTCCAGAAATCGAATGTCTGCAAGAACAAGTGGGACTT<br>TGCCCTCATGGAAAATGGAAGTGCTGCACAGAAGTG	KFO88553.1
Ceyx cyanopectus	VYZU01073949.1	TTGCCCAAAGACACCCTACGTTGTCTTGGATACCACGGGTTCTGCTTCCGTCC<br>AAAATCCTGCCCAGAGCCATTTGCTGCCTTTGGAACTTGCTCTTGGCGTCACA<br>AAACATGCTGCATAGACACGACATCAAAATACCGCACTTGCCAAGATGAGG<br>GGGGTCACTGTGTACCTCAAAAAATCAAATGTGTGCAAGAACAAGTGGGACT<br>TTGCCCTCACAGAGAATGGAAGTGCTGCACAGAAGTA	NXY89543.1
Furnarius figulus	VYZD01000216.1	TTGCCCAAAGACACCCTGCGTTGTTTGGAATACCACGGCTACTGCTTCCATTT<br>GAAATCCTGCCCAGAGCCATTTGCTGCGTTTGGAACTTGCTACCGGCGTCGG<br>AAAACCTGCTGTGTGGACACAACATCCATCTCCCACATTTGTCGAGAGGAGG<br>GGGGTCACTGTGTAGCCCCACACATCAGGTGCCTGCAAGAACAAGTGGGACT<br>CTGCCCTCGCAGGGGATGGAAGTGCTGCTCAGAATTG	NWR87765.1
Pseudopodoces humilis	XM_005528784.1	TTGCCCAGAGACACCTTGCGTTGTTTGGAATACCACGGCTACTGCTTCCATCT<br>GAAATCCTGCCCAGAGCCGTTTGCTGCCTTTGGAACTTGCTACCGGCGCCGC<br>AGGACCTGCTGCGTTGACACGACATCCAACTTCCACATCTGCCAAGATGAGG<br>GGGGCCACTGTGTGCCCCCAGAAATCAAATGTCTGCAAGAGCAAGAGGGAC<br>TCTGCCCTCGCAGAGGATGGAAGTGCTGCACAGAAGTG	XP_005528841.1
Zosterops hypoxanthus	VWYL01016114.1	TTGCCAAGGGACACCCAGCGCTGTTTGGAACACCACGGTTACTGCTTCCACC<br>TGAAATCCTGCCCGGAGCCGTTTGCTGCCTTTGGAAGCTGCTACCGGCGCCG<br>GAGGACCTGCTGTGTTGACACGACATCCAACTTCCATGCCTGCCAGGATGAG<br>GGGGGCCACTGTGTGTCCCCAGAAATCAGATGTCTGCAAGAGCAAGAGGGA<br>CTCTGCCCCCGCAGAGGATGGAAGTGCTGCTCAAAAGTG	NXR36414.1
Catharus ustulatus	XM_033056724.1	TTGCCCAGGGACACCTCACGTTGTTTGGAACACCACGGTTACTGCTTCCACCT<br>GAGATCCTGCCCGGAGCCGTTCGCCGCCTTCGGGACCTGCTACCGGCGCCGC<br>AGGACCTGCTGTGTGGACACAACATCCAACTTCCACATCTGCCAGCACGAGG<br>GGGGCCACTGTGTGCCCCCAGAAATCAGATGTGTGCAGAAGCAGGAGGGGC<br>TCTGCCCTCGCAGAGGATGGAAGTGCTGCACAGAAGTG	XP_032912615.1
Taeniopygia guttata	XM_002186628.3	TTGCCCAGGGACACCCTGCGTTGTTTGGAATACCACGGCTACTGCTTCCACCT<br>GAAATCCTGCCCAGAGCCGTTCGCTGCCTTTGGAACCTGCTATCGGCGCCGC<br>AGGACCTGCTGCCTTGACACGACATCCAACTTCCACATCTGCCAAGATGAGG<br>GGGGCCATTGTGTCCCCCCAGAAGTCAGATGTCTGCAGGAGCAAGAGGGACT<br>CTGCCCTCGCAGAGGATGGAAGTGCTGCACAGAAGTG	XP_002186664.2
