# Supplementary figures and images for: The Two Domains of the Avian Double-β-Defensin AvBD11 Have Different Ancestors, Common with Potential Monodomain Crocodile and Turtle Defensins
Source: Biology (Basel). 2022 Apr 30;11(5):690. doi: 10.3390/biology11050690 (PMC9138766; doi:10.3390/biology11050690)

**Figure S1. Full (untrimmed) MSA of monodomain AvBDs and OvoDs**

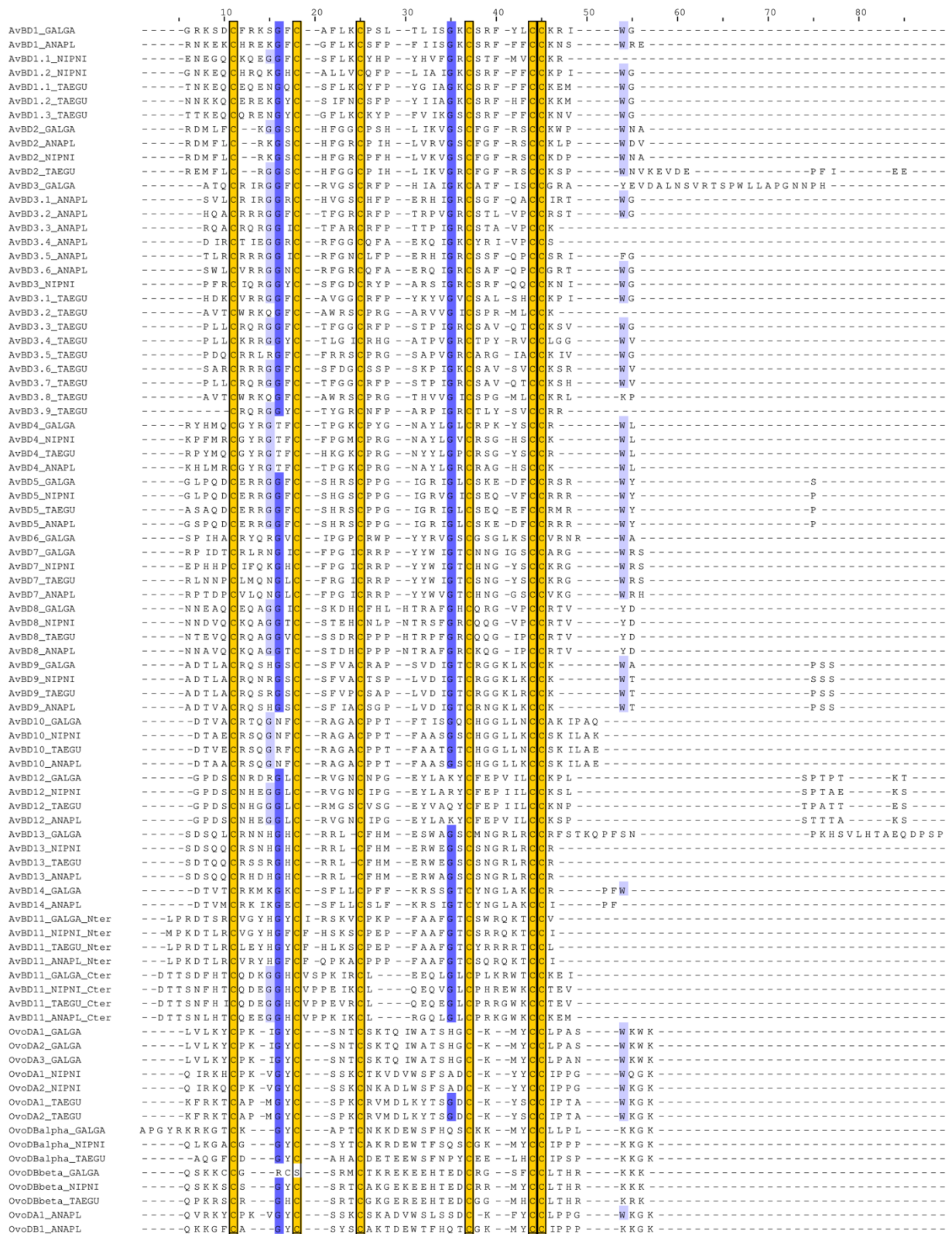

Supplement: Supplementary file 1 [file biology-11-00690-s001.zip › Figure S1.pdf]
