# Supplementary material for: The Two Domains of the Avian Double-β-Defensin AvBD11 Have Different Ancestors, Common with Potential Monodomain Crocodile and Turtle Defensins
Source: Biology (Basel). 2022 Apr 30;11(5):690. doi: 10.3390/biology11050690 (PMC9138766; doi:10.3390/biology11050690)

Figure S3. Full (untrimmed) MSA of monodomain AvBDs and crocodile beta-defensins.

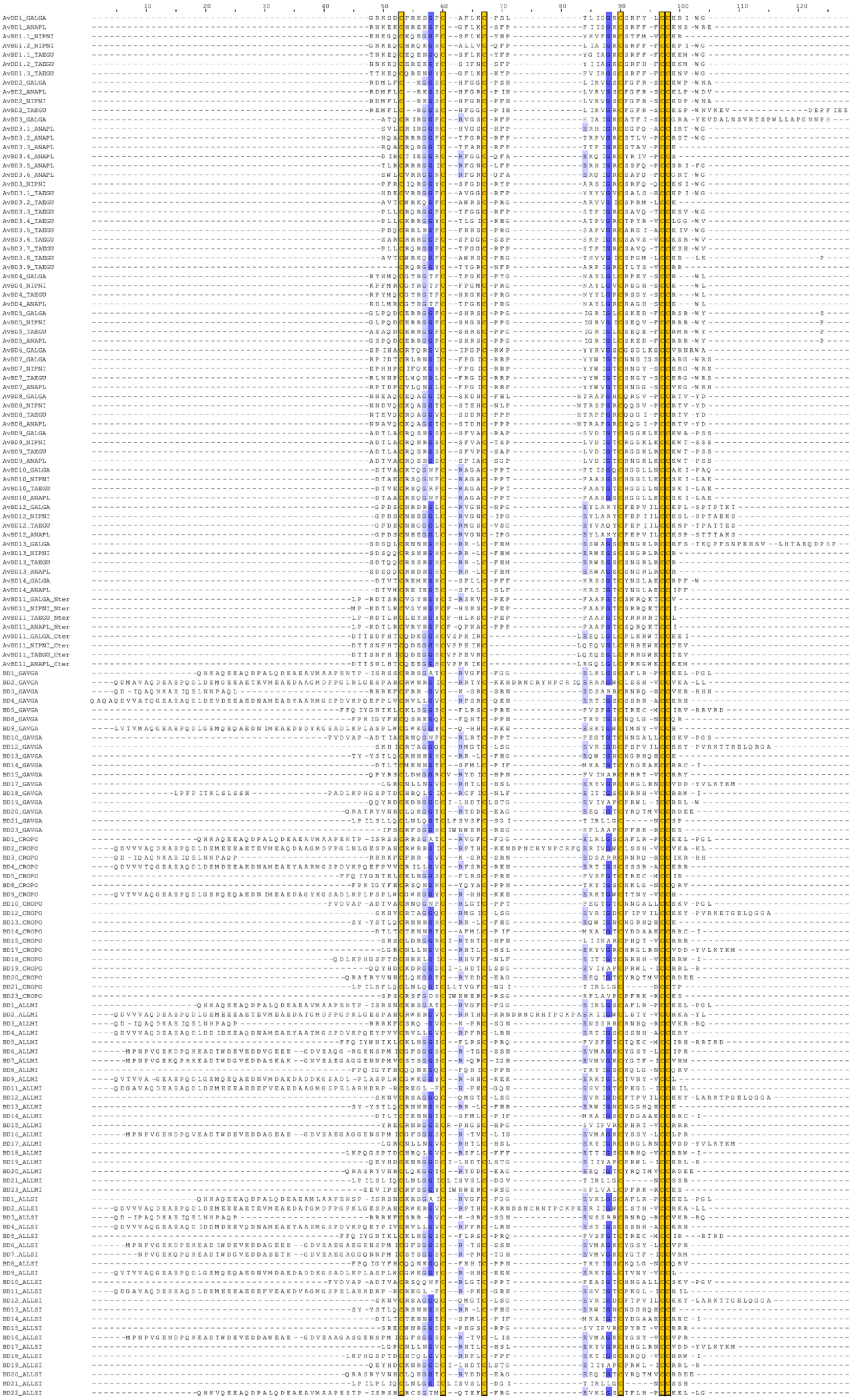

Supplement: Supplementary file 1 [file Figure S3.pdf › Figure S3.pdf]
